# Cajaninstilbene Acid and Its Derivative as Multi-Therapeutic Agents: A Comprehensive Review

**DOI:** 10.3390/molecules29225440

**Published:** 2024-11-18

**Authors:** Wen Hou, Lejun Huang, Jinyang Wang, Walter Luyten, Jia Lai, Zhinuo Zhou, Sishuang Kang, Ping Dai, Yanzhu Wang, Hao Huang, Jinxia Lan

**Affiliations:** 1 Jiangxi Province Key Laboratory of Pharmacology of Traditional Chinese Medicine, School of Pharmacy, Gannan Medical University, Ganzhou 341000, China; twenhou@gmu.edu.cn (W.H.);; 2 School of Rehabilitation, Gannan Medical University, Ganzhou 341000, China; 18379811337@163.com; 3 Department of Biology, Animal Physiology and Neurobiology Section, KU Leuven, 3000 Leuven, Belgium; 4 School of Public Health and Health Management, Gannan Medical University, Ganzhou 341000, China

**Keywords:** *Cajanus cajan*, cajaninstilbene acid, antibacterial activities, anti-tumor, anti-inflammatory

## Abstract

Pigeon pea (*Cajanus cajan* (L.) Millsp.) is a traditional Chinese medicinal plant widely utilized in folk medicine due to its significant pharmacological and nutritional properties. Cajaninstilbene acid (CSA), a stilbene compound derived from pigeon pea leaves, has been extensively investigated since the 1980s. A thorough understanding of CSA’s mechanisms of action and its therapeutic effects on various diseases is crucial for developing novel therapeutic approaches. This paper presents an overview of recent research advancements concerning the biological activities and mechanisms of CSA and its derivatives up to February 2024. The review encompasses discussions on the in vivo metabolism of CSA and its derivatives, including antipathogenic micro-organisms activity, anti-tumor activity, systematic and organ protection activity (such as bone protection, cardiovascular protection, neuroprotection), anti-inflammatory activity, antioxidant activity, immune regulation as well as action mechanism of CSA and its derivatives. The most studied activities are antipathogenic micro-organisms activities. Additionally, the structure–activity relationships of CSA and its derivatives as well as the total synthesis of CSA are explored, highlighting the potential for developing new pharmaceutical agents. This review aims to provide a foundation for future clinical applications of CSA and its derivatives.

## 1. Introduction

Natural products (NPs) are regarded as important resources in drug discovery [1,2]. In comparison with chemically synthesized substances, some NPs offer advantages such as high bioavailability, reliability, low toxicity, and minimal side effects, yielding numerous bioactive compounds that hold significant medical value [3,4]. Pigeon pea [*Cajanus cajan* (L.) Millsp.], belonging to the leguminosae family, is widely distributed across tropical and subtropical regions [5]. Originating from India and found in southern and southwestern China, pigeon pea ranks as the world’s sixth-largest edible legume and is a unique woody leguminous plant [6,7]. Beyond its nutritional significance, pigeon pea offers diverse applications, prominently featuring in traditional medicine across China, India, and other nations [8,9]. According to the “Chinese Materia Medica”, pigeon pea seeds, roots, and leaves are utilized in medicinal contexts. For example, *Cajanus cajan* is indicated in the relief of pain in traditional Chinese medicine and as a sedative [10]. It has also been demonstrated to treat the ischemic necrosis of the caput femoris, aphtha, bedsore, and wound healing. In addition, it is used as an infusion or tea preparation against various skin diseases, including bedsores, oral ulcers, and measles, as well as urinary tract infections, menstrual disorders, genital irritations, hepatitis, diabetes, dysentery, and so on in traditional medicine in West African and India [11,12]. Active components found in pigeon pea leaves primarily include stilbenes and flavonoids, including cajaninstilbene acid (CSA), longistylin A (**2**), longistylin C, and pinostrobin [13].

**Figure 1:**
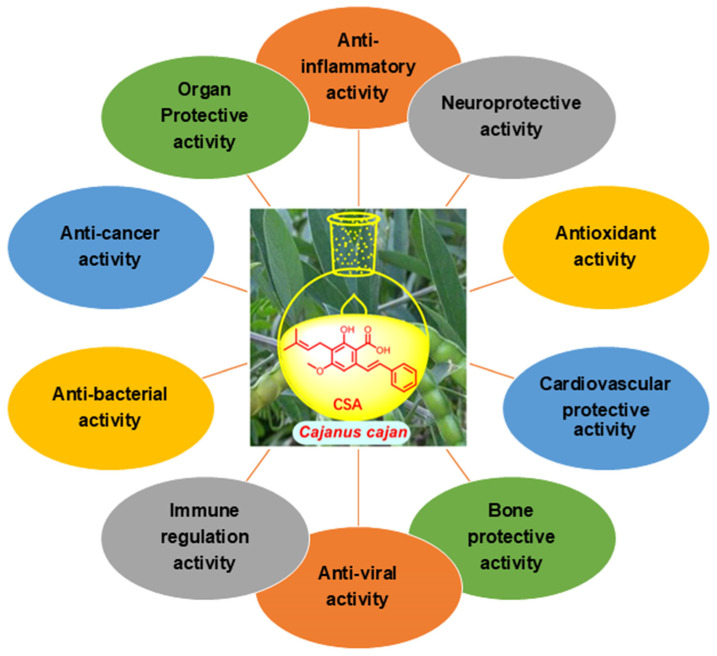
Picture of *Cajanus cajan*, structure of cajaninstilbene acid (CSA), and the bioactivities of CSA and its derivatives.

CSA (chemical structure is displayed in Figure 1. All the chemical structures in this review are made by Chembiooffice2014) is a stilbene compound derived from pigeon pea leaves. Its chemical name is 2-carboxyl-3-hydroxy-4-isopentenyl-5-methoxystilbene, with a molecular formula C_21_H_22_O_4_ and a molecular weight of 338.4. CSA exhibits high lipophilicity (AlogP of 5.0) [14,15]. The presence of hydroxyl, carboxyl, stilbene, and methoxy groups in CSA contributes to its diverse pharmacological effects, including antioxidant [16], antitumor [17], hypolipidemic [18], and antibacterial properties [19] (Figure 1) (all the pictures in this review are made by Microsoft PowerPoint and Adobe photoshop CS6). This review explores the biological activities and mechanisms of CSA and its derivatives, providing insights into future research and the development of CSA-related compounds.

## 2. Pharmacological Activities

### 2.1. Pharmacokinetic Characteristics and Metabolism of CSA

**Scheme 1:**
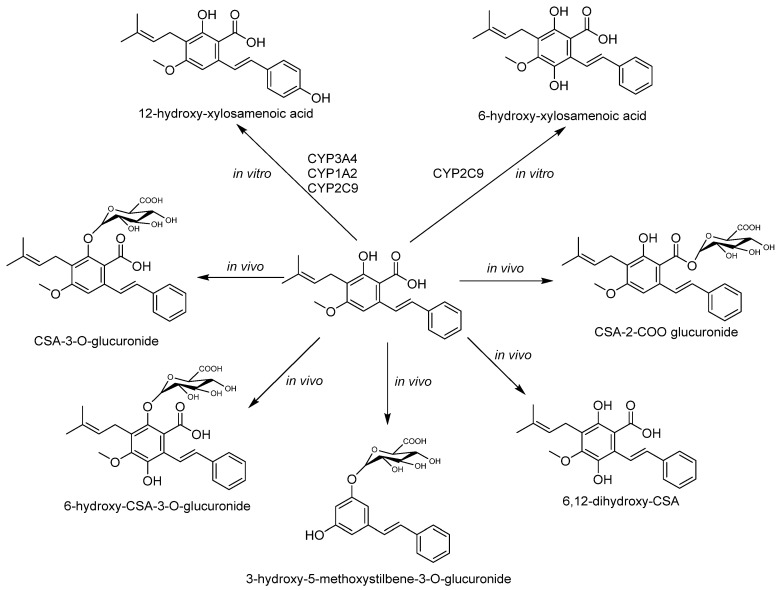
CSA metabolism demonstrated by assays in vivo or in vitro.

By applying liquid chromatography with the tandem mass spectrometry method, researchers found that CSA distributes rapidly and extensively with rapid elimination (T_max_ of 10.7 ± 0.31 min, t_1/2_ of 51.40 ± 6.54 min), particularly in the small intestine, liver, and kidneys after oral administration [20]. Its oral bioavailability is approximately 44.36% [21]. Xin Hua et al. identified two metabolites, M1 (12-hydroxy-xylosamenoic acid) and M2 (6-hydroxy-xylosamenoic acid) (Scheme 1), involved in CSA metabolism using human liver microsomes and recombinant human P450 enzymes in vitro assays. The major CYP450 enzymes responsible for CSA hydroxylation in liver microsomes are CYP3A4, CYP2C9, and CYP1A2. CYP2C9 catalyzes all oxidative metabolite formations, while CYP3A4 and CYP1A2 are involved in M1 formation. CSA reversibly inhibits CYP3A4 and CYP2C9 activity (IC_50_ of 28.3 and 31.3 µM, respectively) [20,21]. Li-Sha Wang et al. reported five rat metabolites of CSA (M1-M5): CSA-3-O-glucuronide, CSA-2-COO glucuronide, 6,12-dihydroxy-CSA, 3-hydroxy-5-methoxystilbene-3-O-glucuronide, and 6-hydroxy-CSA-3-O-glucuronide (Scheme 1). Studies indicate extensive conversion of CSA to its metabolites, particularly glucuronic acid metabolites, primarily through the hepatic metabolism into the systemic circulation, with glucuronide-conjugated and hydroxylated metabolites excreted via bile. M1 (CSA-3-O-glucuronide), formed post-first-pass metabolism, reduces CSA’s oral bioavailability. Furthermore, CSA’s enterohepatic circulation, extravascular distribution, and renal reabsorption characteristics contribute to delayed elimination. Notably, metabolism within the body, rather than absorption, represents the primary limitation to CSA’s oral bioavailability [22,23].

### 2.2. Antitumor Activity

**Figure 2:**
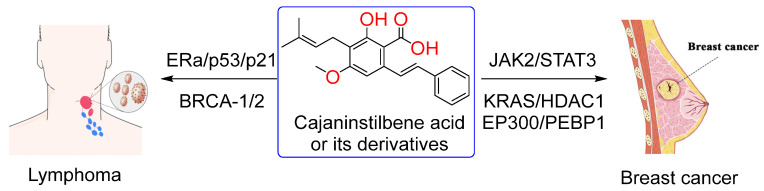
The main regulatory pathways or targets of the antitumor activity of CSA and its derivatives.

The general antitumor activity of CSA is ordinary. Consequently, recent literatures have primarily focused on investigating the anticancer potential of CSA derivatives (Table 1 and Table 2). Studies have shown that CSA and its derivatives are broad-spectrum anticancer agents, demonstrating effective inhibition of tumor cell proliferation, particularly in lymphoma and breast cancer cells (Figure 2).

#### 2.2.1. Antitumor Activity of CSA

Breast cancer is a prevalent malignancy and a leading cause of cancer-related mortality [34]. Hormone receptors such as estrogen and progesterone receptors (ER, PR) and growth factor receptors (HER2, etc.) are crucial prognostic indicators for breast cancer [35]. Given that most breast cancers are hormone-dependent and ER-positive, anti-estrogen therapies are pivotal in improving patient prognosis. CSA is anticipated to mimic tamoxifen and exert anti-ERα-positive effects in breast cancer by binding to and inhibiting ERα. Upon binding of 17β-estradiol (E2) to estrogen receptor-α (ERα), a conformational change occurs that activates transcription factors and alters gene expression [36]. The E2–ERα complex can promote breast cancer growth [37,38], underscoring the role of antiestrogen therapy in breast cancer treatment. CSA exhibited greater toxicity and specificity towards ERα-positive MCF-7 cells (IC_50_ of 61.25 ± 2.67 µM) compared to ERα-negative MDA-MB-231 cells (IC_50_ of 175.76 ± 19.59 µM). Furthermore, CSA showed cytotoxicity against tamoxifen-resistant MCF-7 (MTR-3) cells (IC_50_ of 188.22 ± 38.58 µM) by downregulating ERα protein expression both in vitro and in vivo. Combination treatment of CSA (100 µM) with tamoxifen (0.3 µM and 1 µM) synergistically enhanced cytotoxicity and upregulated p53 protein expression. In vivo studies revealed that CSA (tumor inhibition rates were 43 and 66% at 15 and 30 mg/kg dosages, respectively) exerted stronger inhibitory effects on MCF-7 xenografts in nude mice compared to the positive control cyclophosphamide (20 mg/kg). CSA treatment did not affect the weight of tumor-bearing nude mice, whereas cyclophosphamide-induced slight weight loss and one mouse died. CSA’s structural similarity to estrogen and its shared binding site with 17β-estradiol and tamoxifen on ERα confer antiestrogenic properties, suggesting its potential as a novel antiestrogenic agent in breast cancer treatment [24].

Targeting breast cancer-associated receptors (ER, PR, EGFR, etc.) influenced cancer pathways involved in apoptosis, cell cycle regulation, and DNA damage. CSA inhibits the growth of ER-positive MCF-7 cells by inducing DNA damage and modulating cell cycle-related regulators like the tumor suppressor p21 and BRCA-1/2 genes, resulting in cell cycle arrest and apoptosis [39,40]. CSA induced G2/M phase arrest and apoptosis both in vitro (8.88–14.79 µM in MCF-7 cells) and in vivo (15 or 30 mg/kg in MCF-7-xenografted nude mice), whereas cyclophosphamide affected the S phase instead of G2/M. CSA enhanced mitochondrial depolarization and caspase 3 activity in a dose-dependent manner, decreased BcL-2, and increased Bax expression in MCF-7 cells. It primarily impacted BRCA1-related DNA damage response pathways and chromosome replication control during the cell cycle. It downregulated BRCA-1/-2 and upregulated p21 expression [25]. CSA also exhibited significant cytotoxic effects on HeLa, CaCo-2, and MCF-7 cells, with IC_50_ values ranging from 39 to 80 µg/mL, 32 to 80 µg/mL, and 42 to 80 µg/mL, respectively [26]. Nadire Ozenver et al. identified serine-threonine kinase WNK3 (also known as lysine-deficient protein kinase 3) as the most likely target of CSA using whole kinase group methodology [17].

#### 2.2.2. Antitumor Activity of CSA Derivatives

##### Antitumor Activity of Natural Derivatives of CSA

**Figure 3:**
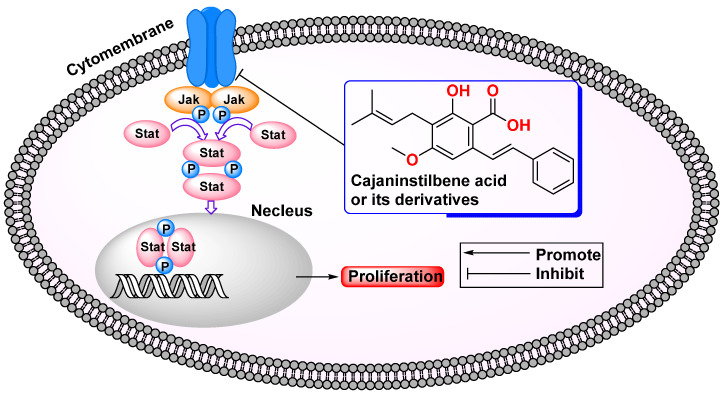
JAK/STAT pathway related to the antitumor activity of CSA or its derivatives.

Longistylin A (**2**) and longistylin C (**3**), two compounds isolated from the leaves of pigeon pea, are natural derivatives of CSA (Table 2) and have broad-spectrum antitumor activity. J.S. Ashidi et al. reported that longistylin A (**2**) (2.4–20.04 µM) and longistylin C (**3**) (5.8–18.3 µM) were cytotoxic to six cancer cell lines (MCF-7, COR-L23, C32, HepG2, 16HBE4o, and AR42J-B13) (Figure 3), and vincristine (IC_50_ of 0.2–0.6 nM) was used as a positive control [30]. Xing-Yue Ji et al. reported that longistyline A (IC_50_ of 24.65 µM) and CSA (IC_50_ of 19.14 µM) have weak anti-proliferation activity on human liver cancer Bel-7402 cells (the positive control is vincristine, IC_50_ of 0.005 µM) [29]. En-Nian Li et al. reported that longistylin C (IC_50_ of 14.4~29.6 µM) significantly inhibited cell proliferation in all seven tumor cell lines (MDA-MB231, HeLa, HepG2, SW480, A549, NCI-H460, and NCI-H1299). It also had a weak inhibitory effect on five tumor cell lines (IC_50_ of 44.9~78.3 µM) except for HeLa and SW480. The positive control drug was tri-*O*-methylresveratrol (IC_50_ of 3.0~46.0 µM) [28].

Non-Hodgkin lymphoma (NHL) includes a diverse group of malignancies arising from B and T lymphocytes [41]. Compounds that selectively induce apoptosis in these cells are crucial for treating this disease. B10 (**4**), which is isolated from *Cajanus cajan*, effectively inhibited the proliferation of non-Hodgkin lymphoma (NHL) Raji cells by inducing endogenous apoptosis and arresting cells in the S/G1 phase. B10 (**4**) (12, 18, and 24 µM, IC_50_ of 18 µM) demonstrated dose- and time-dependent inhibition of Raji cell proliferation. It also reduced cyclin D1 expression by enhancing phosphorylated γ-H2AX levels, inducing DNA damage and cell cycle arrest. Together, B10 (**4**) induced apoptosis in lymphatic Raji cells by modulating the PI3K-mediated JAK2/STAT3 pathway and the BTK-mediated KRAS/HDAC1/EP300/PEBP1 axis (Figure 3) [31,32].

Jia-Zhong Cai et al. first reported naturally occurring ether derivatives 3-monochloro-1,2-propylene glycol (3-MCPD), cajanstilbene H (**5**), which could promote osteoblast differentiation in hMSC, showing the potential to alleviate osteoporosis. Moreover, cajanstilbene H (**5**) showed moderate inhibition of six human cancer cell lines (NCI-H460, PC-3, MCF-7, HeLa, HCT-15, and KB-V1) (IC_50_ of 21.42–25.85 µM), with paclitaxel (IC_50_ of 0.021–0.225 µM) used as a control [42]. Amorfrutin B (**24**), exhibited moderate cytotoxic activity against PANC-1 cells (IC_50_ of 12 µM) under glucose starvation conditions, as reported by Tadafumi Fujita et al. [33].

c-Myc (MYC) is a carcinogenic transcription factor closely linked to cancer cell reprogramming and resistance to chemotherapy [43,44]. Overexpression of c-Myc in tumor cells correlates with the development and progression of various cancers, activated through multiple mechanisms [45,46]. CSA and its derivatives, CSA 6 (**6**) and CSA 19 (**7**) (Table 3), effectively inhibited c-Myc in breast cancer cells [47,48]. CSA (EC_50_ of 15.48 ± 6.84 µM) and its derivatives CSA 6 (**6**) (IC_50_ of 2.96 ± 0.12 µM) displayed inhibitory effects on MCF-7 breast cancer cells. Furthermore, CSA 6 (**6**) (EC_50_ of 5.20 ± 0.27 μM) and CSA 19 (**7**) (EC_50_ of 1.40 ± 0.64 µM) showed lower EC_50_ values compared to known MYC inhibitors 10058-F4 (EC_50_ of 52.63 ± 4.55 µM) and 10074-G5 (EC_50_ of 12.80 ± 0.59 µM) in HEK293 cells transfected with c-Myc-luciferase [27].

Cancer stem-like cells (CSCs) are a unique population within tumors capable of self-renewal and differentiate into multilineages, and are capable of generating new tumor growth [50,51]. Because CSCs drive tumor initiation, metastasis, and resistance to conventional treatments, they contribute significantly to tumor relapse and therapy resistance [52,53,54]. The CSA derivative CSA19 (**7**) effectively reduced collateral sensitivity in CSC-rich MCF-7 cells. According to previous findings by Ean-Jeong Seo et al., CSA6 (**6**) (resistant at 0.92 µM, IC_50_ of 4.98 of 0.64 µM and 5.42 of 23.75 µM, respectively) and CSA 19 (**7**) (resistant at 0.16 µM, IC_50_ of 7.51 ± 1.83 µM and 47.00 of 5.43 µM, respectively) showed stronger cytotoxic effects on CSC-enriched MCF-7 cells (CD44^high^ and CD24^low^) compared to MCF-7 monolayer cells. Compared with MCF-7 monolayer cells, CSC-enriched MCF-7 cells showed greater resistance to the positive controls 5-fluorouracil and docetaxel (1.41 and 9.00 times, respectively). This phenomenon, where resistant cells exhibit increased sensitivity to specific compounds, is known as collateral sensitivity. Derivative (**7**) induced this incidental sensitivity in cancer stem cell-like cells [55].

Lei Zhang et al. reported that derivative 11 (**8**) (structure is showed in Table 3) (IC_50_ of 56.07, 115.85, and 26.80 µM, respectively) displayed stronger inhibitory effects against three test cell lines (HT29, MCF-7, and PA-1) compared to the positive control resveratrol (IC_50_ of 224.45, 347.07, and 53.58 µM, respectively) and tamoxifen (IC_50_ of 91.60, 138.92, and 141.15 µM, respectively). Research indicated that ERα-mediated signaling pathways promote migration and invasiveness of PA-1 cells of malignant immature ovarian teratoma [49]. Derivative 11 (**8**) exhibited robust inhibitory activity against PA-1 tumors and reduced ERα expression in MCF-7 cells. Hence, derivative 11 (**8**) emerges as a promising candidate for treating ovarian immature teratomas [56]. The antitumor activities of CSA derivatives (both natural and synthetic) as well as their mechanism of action are summarized in Table 4.

### 2.3. Systematic Effects and Organ Protection

#### 2.3.1. Neurological Related Diseases

**Figure 4:**
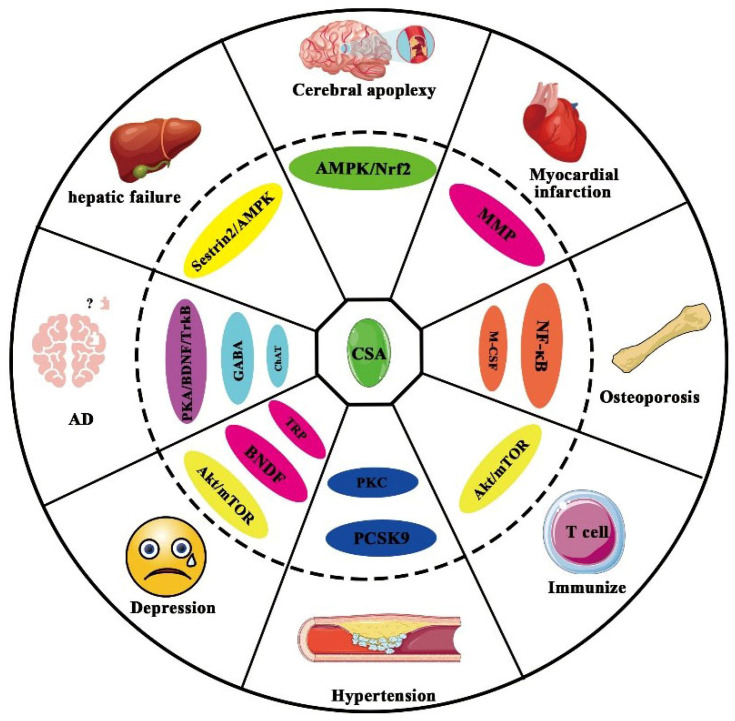
CSA showing systematic and organ protection via the indicated pathways or targets.

Cognitive dysfunction in Alzheimer’s disease (AD) patients correlates significantly with reduced choline acetyl transferase (ChAT) activity and the loss of cholinergic neurons [57,58]. Thus, strategies aimed at reducing reactive oxygen species (ROS) production and enhancing acetylcholine (ACh) production through inhibition of acetylcholinesterase (AChE) activity or increased ChAT activity are pivotal in AD treatment. Studies indicate that estrogen notably ameliorates cognitive deficits and neuronal apoptosis induced by Aβ_25–35_ in mice. Moreover, estrogen can increase superoxide dismutase (SOD) activity and ChAT activity and decrease AchE activity (Figure 4) [59]. Can-Jun Ruan et al. reported that 200 mg/kg of stilbene extracts (predominantly containing CSA) derived from *Cajanus cajan L*. (sECC) significantly improved cognitive deficits and reduced neuronal apoptosis induced by Aβ_25–35_ in mice. They found that 100 mg/kg of sECC could significantly reduce TUNEL-positive cells and no TUNEL-positive cells appeared in the 200 mg/kg of sECC group in the hippocampus of mice. Oral administration of sECC (200 mg/kg) markedly improved the spatial learning disability in the Morris water maze experiment and increased SOD and ChAT activities in both the hippocampus and cortex. By enhancing ChAT activity and antioxidant effects, sECC obviously countered cognitive decline associated with Alzheimer’s disease. Overall, sECC reversed the decline in ChAT activity, boosted SOD levels, and inhibited cell apoptosis through modulation of the cholinergic system, thereby enhancing spatial learning abilities (Table 3) [13].

Amyloid β_1–42_ (Aβ_1–42_) oligomers are pivotal in the early phases of Alzheimer’s disease (AD) and are crucial targets for AD treatment [60,61]. Previous studies have highlighted the neuroprotective effects of CSA [62,63]. Li-Sha Wang et al. also discovered that CSA (7.5 mg/kg, 15 mg/kg, and 30 mg/kg) alleviated learning and memory impairments caused by Aβ_1–42_ oligomers in mice. It also reduced Aβ_1–42_ levels in the hippocampus, dampened microglial activation, and moderated hippocampal astrocyte reactivity. Moreover, CSA reversed dysregulation of Glu and GABA expression, significantly decreased Glu levels at 15 mg/kg, and increased GABA levels at 30 mg/kg. Additionally, CSA downregulated GluN 2B expression by enhancing downstream PKA/CREB/BDNF/TrkB signaling pathways. These results suggest that CSA could be a promising therapeutic agent for early stage AD [64].

Depression has emerged as a significant global health concern [65]. Building on prior research [13], Bao-Ping Jiang et al. hypothesized that CSA could serve as a neuroprotective agent, and thus, a potential antidepressant. Their study revealed that CSA (2.0–16.0 µM) dose-dependently increased the viability of PC12 cells, reduced LDH release, and mitigated apoptosis and DNA fragmentation induced by CORT (a glucocorticoid inducing depression-like behavior in animals). In comparison to the antidepressant dimethacrine (DIM), CSA exhibited significantly stronger protection against CORT-induced apoptosis at equivalent concentrations [66]. Mechanistic investigations demonstrated that CSA restored mitochondrial function by lowering intracellular Ca^2+^ levels in PC12 cells, inhibiting mPTPs opening, and enhancing mitochondrial membrane potential. Furthermore, CSA markedly suppressed caspase 3 activation, prevented cytochrome c translocation to the cytoplasm, and inhibited DFF-45 (DNA fragmentation factor 45, also known as ICAD) degradation. It mitigated DNA fragmentation triggered by caspase-activated deoxyribonuclease (CAD); thus, attenuating mitochondrial apoptosis [67,68,69]. These findings underscore CSA’s potential as a promising antidepressant by safeguarding nerve cells against apoptosis [62].

Ya-Min Liu et al. demonstrated that CSA (1–8 µM) significantly attenuated the detrimental effects of CORT (200 µM) on PC12 cells, preventing neuronal loss and reversing nuclear changes. CSA (2–8 µM) notably reduced LDH release and regulated intracellular calcium levels disrupted by CORT in PC12 cells. At 8 µM, CSA reduced ROS levels and malondialdehyde (MDA) content and restored SOD and CAT activity in CORT-exposed PC12 cells. Furthermore, CSA alleviated ER stress induced by CORT, as evidenced by decreased expression of GRP78, CHOP/GADD153, XBP-1, caspase 12, and caspase 9 in PC12 cells [16,63,70]. However, more detailed molecular investigations are needed to fully understand how CSA exerts its neuroprotective effects.

Previous studies have highlighted the neuroprotective and anti-inflammatory properties of CSA [62,63]. Meng-Di Zhang et al. reported that CSA (15 mg/kg) increased sucrose preference and reduced serum corticosterone levels in mice subjected to chronic unpredictable mild stress (CUMS), with paroxetine (10 mg/kg) used as a positive control. CSA exerted antidepressant effects by inhibiting HPA axis function and increasing levels of neurotransmitters. Additionally, CSA reversed the decrease in hippocampal ACh in CUMS mice, indicating the potential for improving cognitive impairment in animal models of depression [71,72].

**Figure 5:**
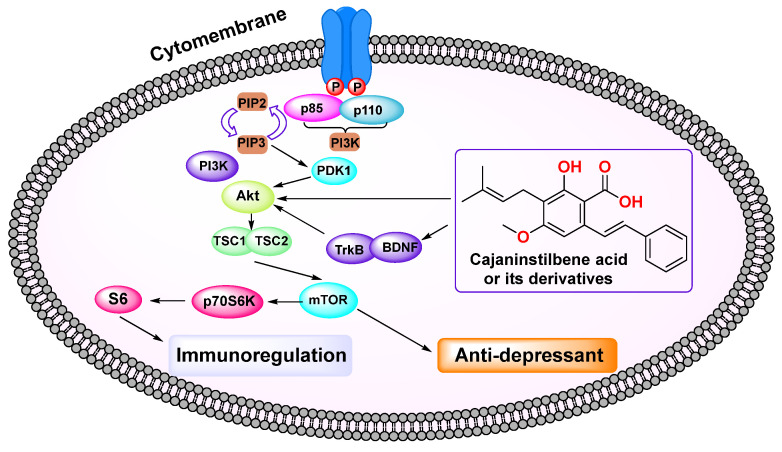
PI3K/AKT/mTOR pathway related to the neuro-related diseases alleviation effect of CSA.

One year later, further investigations demonstrated that CSA (30 or 60 mg/kg) significantly decreased the immobility duration of CUMS mice in both the tail suspension test (TST) and forced swimming test (FST). It (15 or 30 mg/kg) effectively reversed CUMS-induced depression-like behaviors in mice over 3–6 weeks, including increased sucrose preference and reduced feeding latency. The severity of depressive symptoms is strongly linked to reduced tryptophan (TRP) levels [73], and activation of the KYN pathway (KP) [74]. Elevated TRP and BDNF have been associated with enhanced mTOR activation [75,76]. CSA significantly reversed the up-regulation of the KP, reduced kynurenic acid (KYNA) expression, promoted the 5-HT pathway in the cerebral cortex, and thus, increased TRP levels. It also increased BDNF, PSD-95, p-Akt/Akt, and p-mTOR/mTOR levels, suggesting that CSA exerted an antidepressant effect by modulating TRP metabolism, enhancing BDNF expression, and activating the Akt/mTOR pathway (Figure 5), thereby facilitating the synthesis of the postsynaptic protein PSD-95 [77].

Enhancing the brain tissue’s ability to resist oxidative stress is a critical pathway for neuroprotection [78]. CSA provides protection against cerebral ischemia-reperfusion (I/R) and nerve damage induced by oxidative stress. It (0.1–1 µM) reduced intracellular ROS and MDA levels, increased SOD activity, and blocked cytochrome c release. Therefore, the oxidative stress of SH-SY5Y cells was reduced. Adenosine 5′-monophosphate-activated protein kinase (AMPK) can enhance the expression of antioxidant enzymes by activating Nrf2 in the antioxidant system [79,80]. CSA (0.5 μM and 5 mg/kg) promoted the protein expression of Nrf2, HO-1, and NQO1 and inhibited the expression of Keap1 in t-BHP damaged cells and MCAO/R rats. In addition, Nrf2 inhibitor brusatol weakened the neuroprotective efficiency of CSA in vitro and in vivo. These results suggest that the Nrf2 pathway is key to the antioxidant activity of CSA. Moreover, CSA (0.01–5 µM in vitro and 2.5 or 5 mg/kg in vivo) activated the Nrf2 pathway by promoting AMPK phosphorylation in SH-SY5Y cells and MCAO/R rats and enhancing the phosphorylation of Erk 1/2 and p38 MAPK in SH-SY5Y cells. Thus, CSA’s neuroprotective effect involves reducing oxidative stress and mitochondrial dysfunction via AMPK/Nrf2 pathway activation. CSA shows potential as a neuroprotective treatment for ischemic stroke [81].

#### 2.3.2. Cardiocerebral Vascular System Protection

Hypercholesterolemia, atherosclerosis, and cardiovascular disease are closely linked to plasma levels of low-density lipoprotein cholesterol (LDL-C) [82,83,84,85]. Qing-Feng Luo et al. found that sECC (200 mg/kg), containing CSA (**1**), longistylin A (**2**), longistylin C (**3**), and reduced body weight and the atherosclerosis index in high-fat model mice. It also decreased total cholesterol, triglyceride, and LDL-C levels in both serum and liver. Additionally, sECC enhanced serum SOD activity and upregulated mRNA expression of HMG-CoA reductase, CYP7A1 (it is responsible for catalyzing the conversion of cholesterol to bile acids to excrete liver cholesterol), and LDL receptor (it plays a key role in clearing LDL-C from plasma and maintaining cholesterol balance [86]). Mechanistic investigations revealed that sECC lowered body cholesterol levels by boosting liver LDL-C absorption and promoting cholesterol conversion into bile acids. This suggests that sECC holds promise for reducing blood cholesterol levels and preventing atherosclerosis [87,88]. Proprotein convertase subtilin/kexin type 9 (PCSK9) plays a crucial role in regulating plasma LDL-C levels. Elevated PCSK9 levels decrease LDLR protein in liver cells, thereby increasing plasma LDL-C and cardiovascular disease risk [89,90,91]. According to the results presented by Heng-Yuan Chang et al., CSA reduced PCSK9 gene expression by inhibiting nuclear HNF-1α protein levels in HepG2 cells. This led to increased LDLR protein levels and enhanced LDL uptake [18].

Dong-Mei Zhang et al. observed that CSA (0.1–10 µM) induced relaxation of isolated rat renal arteries in vitro by blocking calcium entry into nifedipine-sensitive Ca^2+^ channels (nifedipine, 100 nM, an L-type calcium channel blocker, served as a positive control), suggesting that CSA could potentially mitigate diseases characterized by increased vascular smooth muscle tone. However, further research is required, particularly in animal models of hypertension and other vascular conditions [92]. Myocardial ischemia-reperfusion is a leading cause of myocardial infarction, where timely reperfusion can exacerbate oxidative stress injury and lead to cardiomyocyte apoptosis [93,94]. He-Yi Liu et al. explored CSA’s protective effects on cardiomyocytes and its mechanisms. They observed that CSA enhanced the survival of hypoxic H9c2 cardiomyocytes and reduced cytoplasmic LDH release. CSA mitigated oxidative stress by suppressing ROS and MDA production while enhancing SOD and CAT activity in H9c2 cardiomyocytes. Moreover, CSA preserved mitochondrial function by reducing intracellular Ca^2+^ levels, maintaining matrix metalloproteinase (MMP) integrity, and preventing mPTP opening. Additionally, CSA inhibited apoptosis in H9c2 cardiomyocytes by increasing BcL-2 and decreasing Bax, caspase 3 levels. These findings highlight CSA’s potential as a promising therapy for myocardial infarction [95].

#### 2.3.3. Cartilage Protection

Osteoporosis, which results in reduced bone mass, is characterized by an imbalance in bone homeostasis leading to decreased bone formation by osteoblasts and increased osteoclast activity [96,97]. Postmenopausal osteoporosis is categorized as primary osteoporosis. Yuan-Yuan Zheng et al. reported that sECC (200 mg/kg) reversed the increase in serum concentrations of follicle-stimulating hormone (FSH) and luteinizing hormone (LH) in the bone loss model of ovariectomized rats, reducing their concentrations by 11.5% and 15.2%, respectively, but did not affect the level of 17β estradiol (E2). Furthermore, sECC had no irritating effect on the uterine tissue and did not increase uterine weight. In addition, it (50, 100, and 200 mg/kg) increased the number of trabeculae in a bone loss model of ovariectomized rats, improved trabecular structure, inhibited bone loss at the femoral head in a dose-dependent manner, and sECC (100 and 200 mg/kg) improved bone loss in the femur by 30 and 60%, respectively. It did not present the risk of uterine and breast cancer that might be associated with estrogen replacement therapy [98]. In a subsequent study, groups found that CSA (0.001, 0.01, and 0.1 µg/mL) promoted the proliferation of HOS TE85 osteoblasts, stimulated cellular collagen formation, and inhibited osteoclast formation (22.8% inhibition at 0.1 µg/mL). These findings indicate that sECC’s effect on estrogen-deficient bone loss involves enhancing osteoblast function and suppressing osteoclast formation [99].

Chang-He Liu et al. reported that pigeon pea paste containing CSA components had anti-osteoporosis effects. Pigeon pea paste increased the content of bone calcium and phosphorus and the rate of cementation in rats with RA-induced osteoporosis and effected reducing bone loss. Moreover, pigeon pea paste significantly increased the trabecular area and trabecular volume and improved the pathological changes in the bone tissue of osteoporotic rats treated with Xianling Gubao (300 mg/kg). High and low doses of pigeon pea paste did not influence the body weight of rats. In addition, it reduced the spleen index and increased the adrenal index [100].

**Figure 6:**
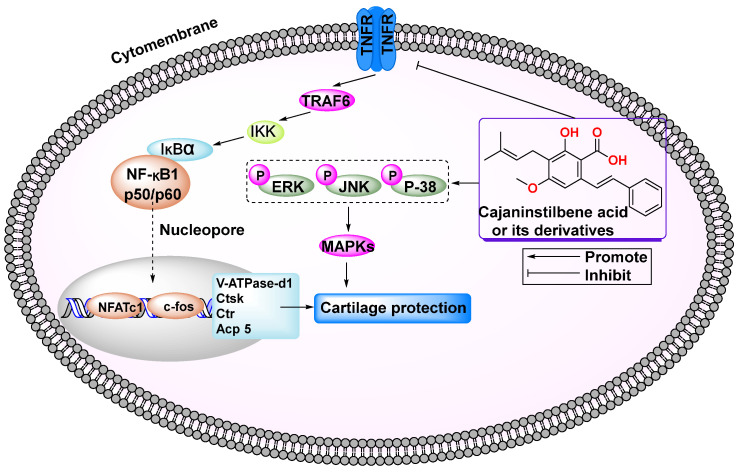
NF-κB and MAPK pathway related to the cartilage protection and anti-inflammation activity of CSA.

You-Qiang Sun et al. discovered that CSA (2.5–10 µM) effectively suppressed RANKL-induced osteoclast formation, bone resorption, and expression of specific genes in a dose-dependent manner. It selectively targeted mature osteoclasts without affecting osteoblast viability. Osteoclastogenesis and function are regulated by macrophage colony-stimulating factor (M-CSF) and receptor activator of NF-κB ligand (RANKL) (Figure 6) [101]. CSA inhibited RANKL-induced NF-κB activation, IκBα protein degradation, and ERK phosphorylation in bone marrow mononuclear cells (BMMs). Moreover, it reduced NFATc1, c-Fos, and V-ATPase-d1/2 proteins level, suppressed ROS activity, and inhibited calcium oscillations in RANKL-stimulated osteoclasts. CSA showed efficacy in reducing ovariectomy-induced bone loss by decreasing osteoclast number and activity in vivo [102]. Jia-Zhong Cai et al. reported that CSA (1–4 µM) promotes osteoblast differentiation in hMSC in a dose- and time-dependent manner [42]. These findings highlight CSA’s potential as a therapeutic agent for osteolytic bone diseases.

#### 2.3.4. Immunoregulation

Natural killer (NK) cells are crucial effector lymphocytes of the innate immune system, responsible for early control of intracellular pathogen infections and immune surveillance against various tumors [103,104]. NK cells exert cytotoxic effects on target cells through cytokines such as interferon-gamma (IFN-γ) [105,106], making it imperative to explore safe compounds that enhance NK cell activity. Jing Geng et al. reported that the CSA derivative LJ101019C (**9**) (0.1–1 µM) promoted NK cell proliferation and enhanced NK cell cytotoxicity against MIA PaCa-2 cells by increasing IFN-γ secretion. LJ101019C (**9**) (1 µM) increased the proportion of NK cells, elevated intracellular ROS levels, and enhanced mitochondrial mass. Mechanistic studies indicated that it activated the AKT/mTOR signaling pathway by upregulating Kv1.3 protein expression in NK cells. It shows promise as a candidate for enhancing NK cell-based immunotherapy efficacy [107].

#### 2.3.5. Liver Protection

**Figure 7:**
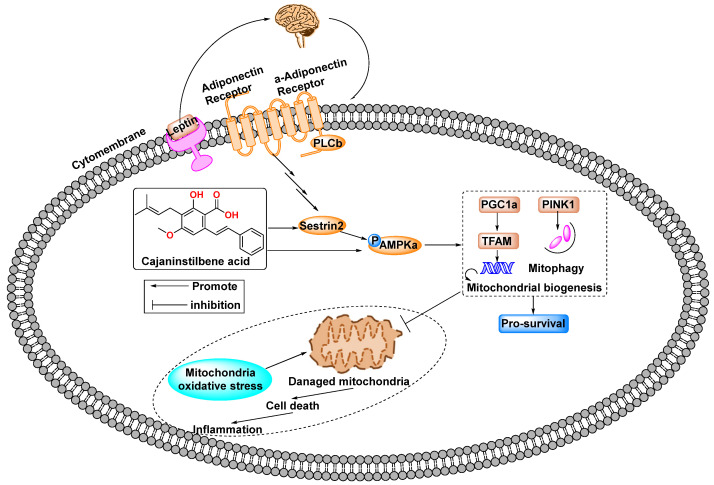
AMPK pathway related to the antioxidant activity of CSA.

Acetaminophen is a widely used over-the-counter drug for pain relief and fever reduction in clinical settings [108]. Overdosing on acetaminophen can lead to liver toxicity and potentially acute liver failure (ALF) [109]. Acetaminophen-induced liver injury (AILI) stands as the leading cause of ALF [110]. However, the efficacy of the current antidote for AILI, N-acetylcysteine (NAC), diminishes significantly following the metabolic activation of acetaminophen [111]. Ming-Zhu Yan et al. studied whether CSA could alleviate acetaminophen-induced hepatotoxicity as a potential new therapeutic strategy. They found that CSA (50 and 75 mg/kg) mitigated liver damage caused by acetaminophen. Levels of alanine aminotransferase (ALT) and aspartate aminotransferase (AST) in serum decreased to 47% (ALT), 37% (AST), and 87% (ALT), 82% (AST), respectively, after 6 h and 24 h of CSA (75 mg/kg) treatment. Histological results showed that CSA significantly weakened the degree of acetaminophen-induced necrosis and apoptosis in area three of the hepatic lobule, and significantly reduced the necrotic area and the number of TUNEL positive cells in mice. CSA also attenuated liver inflammation induced by acetaminophen poisoning. Mechanistic studies revealed that CSA achieved these effects by activating the sestrin2/LKB1/AMPK pathway (Figure 7), thereby preventing AILI [112]. The organs protection and immunoregulation, activities of CSA as well as their mechanism of action are summarized in Table 5.

### 2.4. Activity Against Pathogenic Microorganisms

#### 2.4.1. Antibacterial Activity

##### Antibacterial Activity of CSA and Its Natural Derivatives

In 1982, Cooksey et al. found for the first time that a stilbenate compound isolated from a methanol extract of soybean leaves, had antifungal activity and determined its structure as CSA [113]. Yuan-Gang Zu et al. reported that supercritical fluid extraction (SFE) of *Cajanus cajan* (L.) Huth (the main component of which is CSA) exhibited a significant antibacterial effect. The SFE-CO_2_ extracts showed varying minimum inhibitory concentrations (MIC) ranging from 20 to 2.5 mg/mL, and minimum bactericidal concentrations (MBC) from 390 to 2.5 mg/mL. Particularly noteworthy was their potent antibacterial activity against *S. epidermidis*, *S. aureus*, and *P. vulgaris*, with an MIC as low as 3.9 μg/mL.

Gram-negative bacteria are characterized by an outer membrane surrounding their cell wall, whereas Gram-positive bacteria lack this outer membrane and have a more permeable cell wall. Consequently, SFE-CO_2_ extracts exhibited greater efficacy against Gram-positive bacteria (such as *Staphylococcus* and *Bacillus*) compared to Gram-negative bacteria (including *E. coli* and *Pseudomonas*). This is attributed to the ability of the primary antibacterial components in SFE-CO_2_ extracts to interfere readily with cellular functions [114]. This phenomenon was also noted in the study conducted by Yu Kong et al., who identified cajnuslactone (**10**) (Table 6), a natural derivative of CSA first isolated from plants. It showed strong antibacterial activity with an MIC of 0.031 mg/mL and MBC of 0.125 mg/mL, while CSA possessed an MIC of 0.025 mg/mL and MBC of 0.100 mg/mL against *S. aureus*. Both compounds exhibited moderate activity against *S. epidermidis* and *B. subtilis* compared to antibiotics (penicillin, chloramphenicol, and erythromycin), albeit weaker in potency [115].

Furthermore, in vitro studies demonstrated that SFE-CO_2_ extracts effectively killed *S. aureus* in a time- and dose-dependent manner. The extracts induced *S. aureus* cell death by reducing the expression of *SaeR*. Additionally, SFE-CO_2_ extracts were found to be non-toxic at concentrations up to 0.05 mg/mL but exhibited cytotoxic effects on Raw 264.7 (IC_50_ of 68.9 µg/mL), Vero (IC_50_ of 62.5 µg/mL), and BHK-21 (IC_50_ of 64.1 µg/mL) cells. And the growth inhibition effect on these normal cell lines was significantly weaker than on human breast cancer cells (IC_50_ of 55.7 µg/mL). In vivo studies have demonstrated that SFE-CO_2_ extract (intragastric administration with 30 or 60 mg/kg) can reduce the liver and spleen weight increase caused by *S. aureus* infection in mice. Importantly, these extracts did not display toxicity towards viscera [124].

Sheng-Nan Tan et al. found that CSA had a significant inhibitory effect on both in vivo and in vitro activity of *Enterococcus* (VRE) strains. In vitro studies have shown that CSA (MIC of 0.5–2 mg/mL) can effectively inhibit VRE-sensitive strains and vancomycin-resistant VRE strains. They also found that CSA (1 and 5 mg/kg, intravenously, twice/day, 7 days) and vancomycin (1 and 5 mg/kg, intravenously, twice/day, 7 days) treated V583 strains infected rats had 50, 90, 10, and 30% survival rates. This indicated that CSA had a significant protective effect on VRE-infected mice than vancomycin. In addition, CSA (5 mg/kg, tail vein injection, twice/day, 7 days) could also significantly reduce the number of bacteria in various organs of VRE-infected mice. They also found that CSA could significantly down-regulate the expression level and coding gene of enterococcal phosphotransferase (PTS) protein, including carbohydrate-specific type II transporters such as mannose and sorbitol. These results indicated that CSA inhibited the carbohydrate specificity of type II transporters in the PTS system, thereby inhibiting the transport and uptake of carbohydrates by enterococcus, and obstructing the normal growth and metabolic function of the bacteria, eventually leading to abnormal energy metabolism and death [125].

Colistin (also called polymyxin E), produced by bacteria, is used primarily to treat infectious diseases [126]. However, the emergence of the polymyxin resistance gene *MCR-1* has jeopardized colistin’s role as a last resort against infections caused by multidrug-resistant microbes [127]. Yue Jia et al. discovered that CSA restored sensitivity to polymyxin B in drug-resistant *E. coli*. The combination of CSA (8 µg/mL) and polymyxin B (1 µg/mL) effectively inhibited mcr-1-positive *E. coli* activity. Additionally, combining polymyxin B (5 mg/kg) with CSA (32 mg/kg) effectively combated drug-resistant *E. coli* infections in vivo. The survival rate of mice treated with CSA and polymyxin B combined (80%) was four times higher than that of the polymyxin B alone group (20%). CSA alone did not reduce bacterial load in the liver and spleen. Mechanistically, O12, O17, and O18 on CSA are key binding sites for MCR-1, CSA blocks MCR-1 enzyme activity by binding to critical amino acids in its active site, thereby restoring polymyxin B sensitivity [128].

Jie-Wei Wu et al. reported that longistylin A (**2**) exhibited potent antibacterial activity against methicillin-resistant Staphylococcus aureus (MRSA) (with MIC or MBC values of 1.56 µg/mL) by disrupting bacterial membranes and enhancing permeability. Its bactericidal action was quicker and stronger than that of vancomycin in vivo. It promoted wound healing and closure in MRSA-infected mice, and significantly reduced the weight loss caused by MRSA infection. It also decreased serum levels of TNF-α (from 537.58 to 675.56 pg/mL to 169.96–191.52 pg/mL) and IL-6 (from 238.31 to 322.85 pg/mL to 77.09–97.47 pg/mL). The CC_50_ value of longistylin A (**2**) against RAW264.7 cells was 8.61 ± 0.57 µg/mL, higher than its MIC against MRSA. Therefore, longistylin A (**2**) shows promise in combating MRSA infections [116]. Bai-Lin Li et al. reported that longistylin A (**2**) exhibited the highest effectiveness against *S. aureus* and MRSA, with MIC and MBC values of 1.56 µg/mL. CSA showed an MIC of 6.25 µg/mL and MBC ranging from 6.25 to 12.5 µg/mL. Vancomycin (positive control) had MIC and MBC values ranging from 0.78 to 1.56 µg/mL and 1.56 to 3.12 µg/mL, respectively. Neither longistylin A (**2**) at 1/4 MIC nor CSA at 1/4 MIC exhibited toxicity to HaCaT cells (human keratinocytes). Mechanistically, both compounds at 1/4 MIC inhibited the expression of MRSA adhesion genes (including *icaA*, *icaD*, *clfA*, and *clfB*) and virulence genes (including *AgrA*, *RNAIII*, and *hla*), and suppressed alpha hemolysin production. Consequently, the formation of MRSA biofilms was hindered [19]. Jia-Yan Chen et al. reported that compounds 1–4 (**11–14**), 6 (**15**), and 7 (**16**) (Table 6) isolated from pea plant leaves exhibited potent antibacterial properties. Notably, natural compounds 6 (**15**) possessed an MIC of 0.78 µg/mL, while 7 (**16**) showed MIC values of 3.12 µg/mL and 1.56 µg/mL against *S. aureus* and *B. cereus*, respectively. Positive control vancomycin possessed MIC values of 0.78 µg/mL against both *S. aureus* and *B. cereus* [117].

##### Antibacterial Activity of Synthetic CSA Derivatives

Multidrug-resistant (MDR) bacteria represent a critical public health concern [129]. In recent decades, bacterial pathogens like methicillin-resistant *S. aureus* (MRSA), methicillin-resistant *Staphylococcus* epidermidis, and penicillin-resistant *S. pneumoniae* have increasingly evolved resistance to antibiotics, posing challenges in treating infections caused by these resistant strains [130]. Previous studies have highlighted the effective antibacterial properties of synthetic derivatives derived from CSA [131]. Zhi-Zhong Geng et al. reported that CSA derivatives 5 b (**17**), 5 c (**18**), 5 j (**19**), and 5 k (**20**) (Table 6) displayed strong antibacterial activity against three Gram-negative bacterial strains (*S. aureus*, *S. epidermidis* and *B. subtilis*) and nine methicillin-resistant *Staphylococcus aureus* (MRSA) strains, exhibiting MIC ranging from 0.5 to 8 µg/mL. Particularly, these compounds were more effective against MRSA. The MIC values of compounds 5 b (**17**), 5 c (**18**), 5 j (**19**), and 5 k (**20**) against MRSA reached 0.5 mg/mL, significantly higher than that of CSA (MIC of 8–32 µg/mL) and the positive controls penicillin (MIC of 32–64 µg/mL) and norfloxacin (MIC of 4–16 µg/mL). Compounds 5b (**17**), 5c (**18**), 5j (**19**), and 5 k (**20**) (Table 6) were found to be non-cytotoxic to RAW264.7 cells at 8 µg/mL (the highest MIC value), and the relative survival rate of treated cells exceeded 90%, indicating favorable selectivity indices (SI) [131]. Protein affinity profiling and transcription profiling by Kuo Lu et al. revealed that compound 5b (**17**) targeted the membrane-associated protein PgsA and disrupted the phosphatidylglycerol synthesis pathway [118].

In 2021, Jia-Hui Yu et al. synthesized a new compound, A6 (**21**) (Table 6), based on the previously identified compound 5b (**17**) (Table 6) [118]. This compound was found to disrupt the cell membrane of MRSA, resulting in a 100% inhibition rate at a concentration of 8 µg/mL over 8 h, surpassing the efficacy of the antibiotics ciprofloxacin, rifampicin, and daptomycin (all tested at 40 µg/mL). It enhanced membrane permeability, leading to leakage of intracellular substances such as nucleic acids and ATP. Moreover, at non-inhibitory concentrations, it showed a synergistic effect with piperacillin, significantly reducing the MIC from 528 µg/mL to 32 µg/mL. Administered at dosages of 50, 100, and 200 mg/kg, it notably reduced the sustained bacterial load of MRSA on the skin of mice, with an antibacterial effect comparable to the control vancomycin. At 10 µg/mL, it showed negligible cytotoxicity to mouse RAW264.7 macrophages and human LO-2 hepatocytes, with a cell survival rate above 90% and did not cause damage to mouse organs. Thus, compound A6 (**21**) is considered a potential candidate for treating persistent MRSA infections [119].

Chang Zheng et al. reported that derivatives 6u (**22**) (Table 6) (MIC of 2–8 µg/mL) and 6x (**23**) (MIC of 4–8 µg/mL) displayed the most robust inhibition against *S. epidermidis*, *S. aureus*, and MRSA strains. Derivative 6u (**22**), associated with bacterial RNA polymerase in docking studies, showed the greatest antimicrobial effect on S. epidermidis and MRSA strains, whereas the antimicrobial activity of derivative 6x (**23**) (MIC of 4 μg/mL) against *S. aureus* was twice that of 6u (**22**). Ampicillin (MIC of 2–4 μg/mL, >64 μg/mL) and ciprofloxacin (MIC of 1–16 μg/mL) served as positive agents for inhibition of the activity of *S. epidermidis*, *S. aureus*, and MRSA. Additionally, derivative 6u (**22**) exhibited no significant cytotoxicity to African green monkey kidney cells at concentrations below 10 × MIC [120]. Quorum sensing (QS) system inhibitors of *P. aeruginosa* are designed to reduce bacterial pathogenicity and drug resistance by inhibiting biofilm formation and virulence factor production [132]. The inhibition of PAO1 biofilm formation (20–50%) by the CSA derivative amorfrutin B (**24**) (Table 6) (1–50 µM) was reported by Xing-Jun Xu and co-workers. It inhibited *LAS* and pseudomonas quinolone signal (*PQS)* systems expression and corresponding virulence factors elastase and pyocyanin production in *P. aeruginosa*’s QS systems [122].

Zhi-Xing Huang further investigated CSA analogs, compounds featuring nitrogen-containing heterocyclic rings or electron-absorbing benzene rings, for their potential to inhibit QS and biofilm formation. They identified that compound 3o (**25**) (Table 6) (50 µM) exerted the most potent inhibitory effect on *P. aeruginosa* biofilms and QS, achieving a biofilm inhibition rate of 49.50 ± 1.35% [122]. Similarly to amorfrutin B (**24**) (Table 6) in previous studies, compound 3o (**25**) also suppressed virulence factor production (elastase, pyocyanin, and pyoverdine) by targeting the *LAS* and *PQS* systems of *P. aeruginosa*. Consequently, it represents a promising candidate for combating bacterial pathogenicity and drug resistance [121].

#### 2.4.2. Antiviral Activity

Herpes simplex virus-1 (HSV-1) and -2 (HSV-2) are enveloped double-stranded DNA viruses that commonly cause cold sores, genital ulcers, and potentially fatal encephalitis [133]. According to Yu-Jie Fu et al., CSA has shown effectiveness against these viruses, with IC_50_ values of 0.12 ± 0.09 µg/mL for HSV-1 and 9.460 ± 12 µg/mL for HSV-2. CSA’s inhibition of HSV-1 was comparable to acyclovir (IC_50_ values: 0.097 ± 0.06 µg/mL for HSV-1 and 0.11 ± 0.01 µg/mL for HSV-2). Additionally, CSA showed broader antiviral activity than acyclovir, impacting multiple stages of viral action on cells (adsorption, replication, and release). In vivo studies indicated CSA’s superior anti-HSV activity, with a slightly lower median effective concentration against HSV-1 (65.63 mg/kg) compared to acyclovir (73.62 mg/kg), while its efficacy against HSV-2 (70.28 mg/kg) was less than that of acyclovir (55.44 mg/kg) [134].

Hepatitis C, caused by hepatitis C virus (HCV) infection, leads to chronic liver inflammation, necrosis, fibrosis, and potentially cirrhosis or hepatocellular carcinoma (HCC) in severe cases [135]. Combining direct-acting antiviral agents (DAAs) with host-targeted antivirals (HTAs) enhances therapeutic outcomes and reduces DAA resistance. Xing-Yue Ji et al. reported that CSA (EC_50_ of 3.17 ± 0.75 µM, SI of 40) is a potent new inhibitor of HCV. It showed similar efficacy against drug-resistant and wild-type HCV strains, synergistically inhibiting HCV replication when combined with approved DAA. However, CSA (AlogP of 5.0) exhibits high lipophilicity, potentially impacting its pharmacological properties. Among the designed compounds, compound 2v (**26**) (EC_50_ of 0.33 ± 0.10 µM, SI of 49) showed the strongest inhibitory activity. Compound 15a (**27**) (EC_50_ of 1.42 ± 0.31 µM, SI of 113, ALogP of 3.4) exhibited efficacy comparable to CSA but with lower hydrophobicity. Both CSA and compound 15a (**27**) targeted chondroitin sulfate *N*-acetylgalactosaminyltransferase 1 (CSGalNAcT-1) to inhibit HCV replication. This novel anti-HCV approach holds promise for mitigating drug resistance in HCV treatment [15].

Coronaviruses, which include SARS-CoV, MERS-CoV, and the novel coronavirus (COVID-19), are responsible for respiratory infections [136]. In addition to the highly infectious and deadly SARS-CoV-2 identified in 2019, some less virulent human coronaviruses like HCoV-OC 43 have also caused severe illness, particularly in young children and elderly individuals with chronic conditions [137,138]. CSA (TC_50_ of 38.49 ± 1.85 µM, EC_50_ of 22.22 ± 1.45 µM) and its derivative 1b (**28**) (Table 6) (TC_50_ of 38.49 ± 5.42 µM, EC_50_ of 12.83 ± 0.68 µM) showed significant inhibitory activity against HCoV-OC-43 [123]. Additionally, compound 6 (**29**) (Table 6) (IC_50_ of 4.65 µM) exhibited notable inhibition against the SARS-CoV-2 M^po^ virus, comparable to the positive control ebselen (IC_50_ of 0.96 µM), indicating its potential as a treatment for COVID-19 [117].

#### 2.4.3. Antimalarial Activity

George Duker-Eshun et al. reported strong inhibitory effects of the compounds longistylin A (**2**) (IC_50_ of 34 ± 11 µM) and C (IC_50_ of 19 ± 2 µM), isolated from Cauda, against the chloroquine-sensitive Plasmodium falciparum strain 3D7 [139].

### 2.5. Anti-Inflammatory Activity

Shao-Mei Sun et al. reported that the active ingredient of cajan leaf, CSA, had anti-inflammatory and analgesic effects. CSA (478, 239, 120 mg/kg) reduced the ear inflammation induced by croton oil in mice in a dose-dependent manner, and its anti-inflammatory effect was greater than that of salicylic acid (a positive control drug, 118 mg/kg). In addition, the CSA preparation agent (at 72, 120, and 200 mg/kg) also had a certain analgesic effect (using aconitine as positive at 0.025 mg/kg), which increased the pain threshold of the mice. At the same time, the CSA preparation agent (10 mg/kg) also had an anti-seepage effect and had a significant inhibitory effect on the permeability of capillaries in the abdominal cavity. There were no adverse reactions at the effective doses [140].

**Figure 8:**
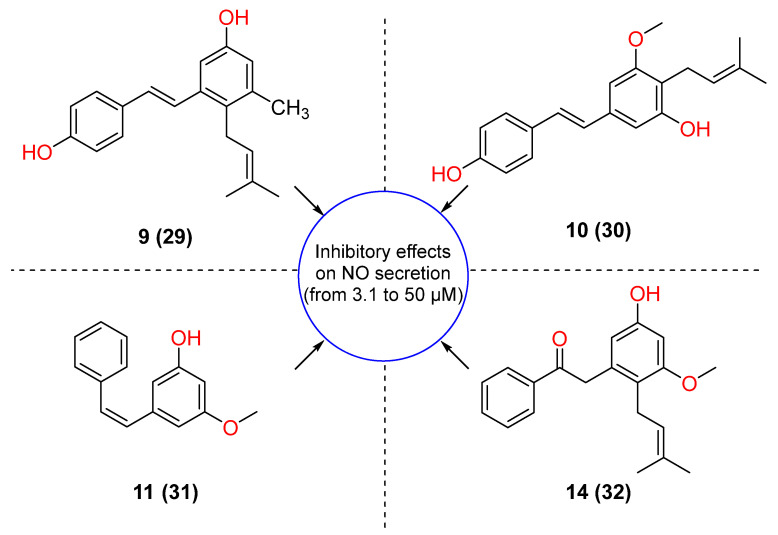
Structure and anti-inflammatory activity of CSA and its derivatives.

Cajanuslactone (**10**) reduced TNF-α and IL-1β levels in LPS-induced RAW 264.7 cells and J774A.1 cells. Additionally, it suppressed LPS-induced TNF-α and IL-1β expression levels to 55.0% and 41.8%, respectively, in rats at a concentration of 20 mg/kg. Positive control dexamethasone (5 mg/kg) reduced TNF-α production by 75.8% and IL-1β production by 63.0%. [141]. According to Ling-Xuan Tan et al. research, CSA derivatives 9 (**29**), 10 (**30**), 11 (**31**), and 14 (**32**) (Figure 8) (3.1–50 μM) isolated from *Cajanus cajan* leaves, exhibited moderate inhibitory effects on LPS-induced NO secretion in RAW264.7 cells [142].

PPARs, a group of transcription factors consisting of PPARα, PPARβ/δ, and PPARγ subtypes, regulate specific target genes when activated by endogenous or exogenous ligands [143]. These factors are pivotal in inflammation, lipid metabolism, cell proliferation, differentiation, cancer, obesity, and energy homeostasis [144]. PPARγ is a negative regulator of inflammation, and increased expression of PPARγ can inhibit the activation of NF-κB and MAPK pathways. CSA and its’ derivative 5c (**18**) exerted their inhibitory effects on the NF-κB and MAPK pathways by enhancing PPARγ activity. CSA (10, 20, and 50 µM) showed a concentration- and time-dependent inhibition of NO release. It reduced NO release by 30.63 ± 2.21% at 20 µM, similar to indomethacin’s inhibition rate of 37.74 ± 3.74% at the same concentration. Interestingly, compared with the positive control indomethacin, CSA (20 µM) significantly inhibited TNF-α and IL-6 more strongly (20 µM CSA and its derivatives had no significant effect on zebrafish cell viability, and the relative cell viability was close to 100%). Moreover, in vivo experiments demonstrated that CSA and derivative 5c (**18**) effectively suppressed the accumulation of neutrophils and macrophages in transgenic zebrafish larvae subjected to tail shear, showing a similar trend to indomethacin. In LPS-stimulated RAW 264.7 cells, treatment with CSA (20 µM) and derivative 5c (**18**) (20 µM) significantly decreased the levels of p-IκBα and p-p65 in the NF-κB pathway, as well as p-ERK (1/2), p-JNK (1/2), and p-p38 in the MAPK pathway (Figure 6). These effects were reversed by the PPARγ inhibitor GW9662 [145]. Therefore, the anti-inflammatory mechanism of CSA and its derivatives involves the expression increasing of PPARγ, thereby inhibiting the activation of NF-κB and MAPK pathways.

Roswitha Schuster et al. reported that CSA extracted from pea showed high transactivation efficiency against PPARγ (positive control, rosiglitazone). CSA had significant anti-inflammatory effects on LPS-stimulated macrophages and significantly inhibited the expression of IL-6 and iNOS (positive control, dexamethasone) [26].

### 2.6. Antioxidant Activity

ROS, such as the superoxide anion (O_2_^−^), hydroxyl free radical (OH), and non-radical compounds, originate from mitochondrial oxidative metabolism and the cytochrome P450 system in hepatocytes [146]. Elevated ROS levels can lead to oxidative damage of cellular biomolecules such as proteins, lipids, and DNA, which are linked to the development of diseases, including atherosclerosis, cancer, and diabetes [147].

Nan Wu et al. reported that CSA exhibited strong antioxidant activity (in the DPPH test system, the IC_50_ value was 302.12 µg/mL). In the beta-carotene-linoleic acid test, the IC_50_ was 321.53 µg/mL, which was greater than that of the positive control ascorbic acid (201.29 µg/mL) and comparable to that of natural antioxidant resveratrol [14]. In a subsequent study (two years later), they found that CSA exhibited enhanced antioxidant properties. The IC_50_ values for scavenging superoxide free radicals, hydroxyl free radicals, nitric oxide, reducing power, and inhibiting lipid peroxidation were 19.03, 6.36, 39.65, 20.41, and 20.58 µM, respectively. CSA also showed concentration-dependent DNA damage protection activity in the range of 7.5–30 µM, surpassing the efficacy of resveratrol (30 µM). Additionally, CSA exhibited xanthine oxidase inhibitory activity with an IC_50_ of 3.62 µM [148]. Jin-Tong Zhao et al. isolated CSA from endophytic fungi *Fusarium solani* (ERP-07), *Fusarium oxysporum* (ERP-10), and *Fusarium proliferatum* (ERP-13) obtained from pigeon pea [*Cajanus cajan* (L.)], exhibiting antioxidant activity nearly equivalent to that of CSA (83%). In experiments to scavenge DPPH free radicals, the inhibition rate of CSA (500 µg/mL) was found to be 80% [149].

**Figure 9:**
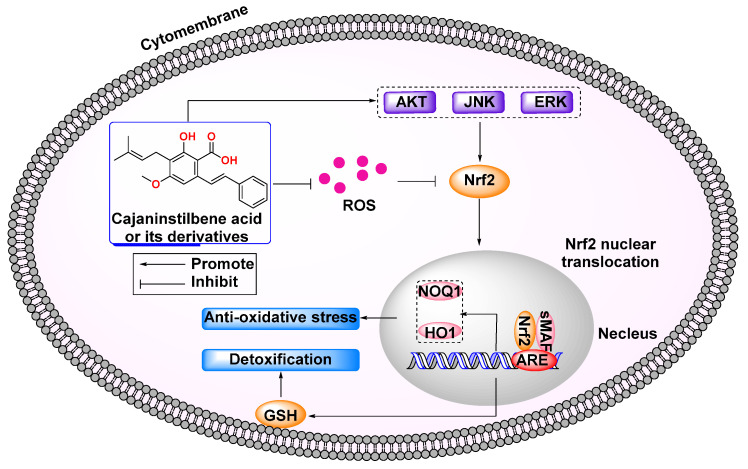
Nrf2-ARE pathway related to the antioxidant activity of CSA.

Additionally, levels of ROS in cells are modulated by genes related to antioxidants such as NAD(P)H: quinone oxidoreductase 1 (NQO 1), heme oxygenase-1 (HO 1), and glutamate-cysteine ligase (GCL) [150,151]. It has been demonstrated that there is a coordinated regulation of the common cis-elements of nuclear factor-erythrocyte 2-associated factor 2 (Nrf2) [152]. In 2013, based on earlier studies [148], Lu Liang et al. conducted further investigations into the antioxidant mechanism of CSA in the HepG2 cell line [153], noting that CSA exerted a dose-dependent suppressive effect on the growth of HepG2 cells (IC_50_ of 17.42 µM). At concentrations of 0.05, 0.1, and 0.5 µM, which were not cytotoxic to HepG2 cells and maintained survival rates above 90%, CSA reduced ROS production in a concentration-dependent manner and triggered the nuclear translocation of Nrf2. The translocation of Nrf2 from the cytoplasm to the nucleus serves as a key activator of phase II antioxidant gene expression, enhances ARE-mediated gene expression activation, and increases the expression of Nrf2-associated antioxidant genes (HO-1 and NQO 1, GCLC, and GCLM-the rate-limiting steps in catalytic GSH biosynthesis). GSH (glutathione), a crucial component of the redox system, plays a significant role in the cellular defense against oxidative damage. It has been proposed that CSA promotes the nuclear translocation and activation of Nrf2 by activating the PI3K/AKT, ERK, and JNK signaling pathways. These findings indicate the critical role of the Nrf2/ARE pathway in regulating the antioxidant effects mediated by CSA (Figure 9) [16].

## 3. Structure–Activity Relationships (SARs) of CSA

**Figure 10:**
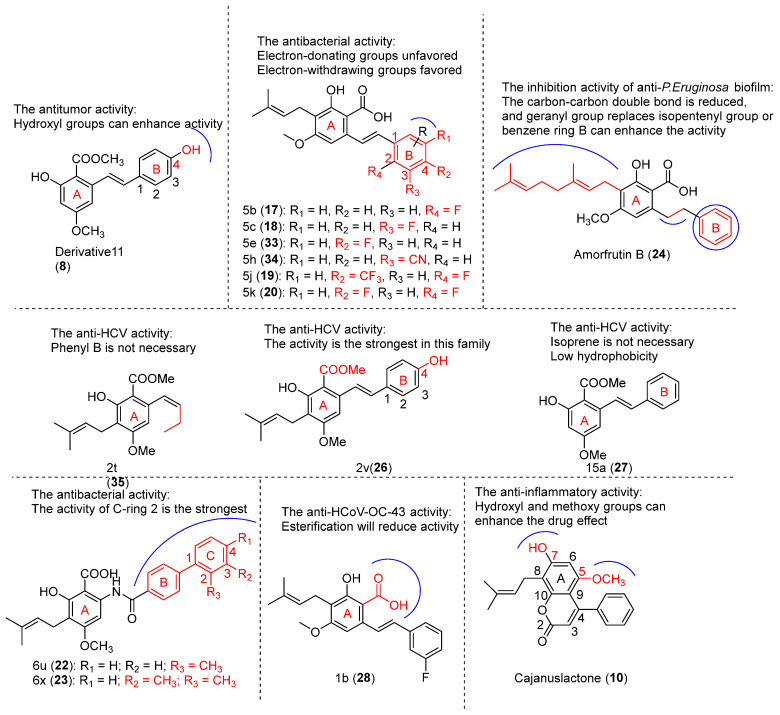
SAR of CSA and its derivatives.

In order to enhance the antitumor, antibacterial, antiviral, and anti-inflammatory effects of CSA, many scholars have conducted in-depth discussions on the structure–activity relationships of CSA based on multiple functional groups, such as stilbene, isopentenyl, carboxyl, hydroxyl, and methoxyl groups (Figure 10).

### 3.1. Studies on the SARs of Antitumor Activity

The SARs of antitumor activity of CSA are seldom reported and the only work is reported by Lei Zhang et al. that derivative 11 (**8**), which involves the introduction of a hydroxyl group at position 4 of the benzene ring B of CSA, significantly enhanced the inhibitory activity against tumor cells (HT29, MCF-7, and PA-1) [56].

### 3.2. Study of the SARs of Antibacterial Activity

In the research on antibacterial activity, Zhi-Zhong Geng et al. observed that removing the styrene and isoprene groups from CSA resulted in reduced or eliminated antibacterial activity. They also found that esterifying the carboxyl group did not enhance antibacterial performance. Therefore, the presence of isopentenyl, free carboxyl, and styrene groups in CSA was crucial for its antibacterial effects. Additionally, introducing electron-withdrawing groups like trifluoromethyl, fluorine, chlorine, and cyano groups into benzene ring B increased antibacterial activity, especially with fluorine atoms, which showed no significant dependency on their number or position. Conversely, introducing electron-donating groups decreased antibacterial efficacy, and substituting benzene ring B with such groups reduced activity. Fluorine-containing compounds (5b (**17**), 5c (**18**), 5j (**19**), and 5k (**20**)) exhibited strong antibacterial activity against Gram-positive bacteria (*S. aureus*, *S. epidermidis*, *B. subtilis*) and methicillin-resistant *S. aureus* (MRSA), with MICs reaching up to 0.5 mg/mL against MRSA [117].

In their study on the biofilm inhibitory activity of the Gram-negative bacterium *P. aeruginosa*, Xing-Jun Xu et al. identified that O-hydroxybenzoic acid or O-hydroxybenzaldehyde in the structure of CSA and its analogs were crucial for biofilm inhibition. They found that reducing the carbon-carbon double bond between the two benzene rings enhanced anti-biofilm activity. Additionally, compounds with geranyl substituents exhibited superior antibiofilm activity compared to those with isopentenyl substituents. Amorfrutin B (**24**), a natural compound lacking a carbon-carbon double bond between the two benzene rings and featuring a geranyl-substituted isopentenyl group, showed significant inhibition of the PAO1 biofilm of *P. aeruginosa* at a concentration of 50 µM, achieving an inhibition rate of 50.3%, indicating moderate antibacterial activity. Xing-Jun Xu et al. argued that reducing the carbon-carbon double bond between the phenyl rings enhanced antibiofilm activity against *P. aeruginosa*. Moreover, Zhi-Xing Huang et al. demonstrated that maintaining the structural integrity of CSA, rather than mimicking QS signaling molecules, was crucial for its biofilm inhibitory activity. Hence, the substitution of geranyl groups for isopentenyl or benzene B was speculated to enhance antibacterial activity against *P. aeruginosa* [122].

Chang Zheng et al. developed a series of CSA derivatives by replacing the trans double bond with an amide bond, which possesses partial carbon-carbon double bond characteristics and good hydrophilicity. This modification allowed the compound to act as both a hydrogen bond donor and acceptor, enhancing affinity with bacterial targets to overcome resistance. They found that derivative 6u (**22**) (2-methyl, 2–4 µg/mL) exhibited the strongest antibacterial activity against *S. epidermidis*, MRSA strains (43,300 and 52,056), the antibacterial activity of derivative 6x (**23**) (2 and 3-methyl, 4 µg/mL) against *S. aureus* was two times higher than that of 6u (**22**). Thus, substituents introduced at the C-ring four position did not enhance antibacterial activity, whether electron-withdrawing or electron-donating groups. Whereas substitution at positions 2 or 3 improved activity, particularly at position 2. Different substituents had varied effects on antibacterial activity (methyl > ethoxy > halogen). Furthermore, simultaneous introduction of two substituents at the 2 and 3 positions of the C-ring enhanced compound activity against sensitive bacteria but diminished efficacy against resistant strains [120].

### 3.3. Studies on the SARs of Antiviral Activity

Since CSA (AlogP of 5.0) is highly lipophilic, Xing-Yue Ji et al. modified the hydrophobic components (the isoprene group and benzene B) of CSA for their anti-HCV study. They found that neither the isoprene group nor benzene B was necessary for inhibiting HCV activity. In their SAR analysis, it was observed that introducing chlorine or fluorine atoms at positions 2 or 4 of benzene ring B enhanced inhibitory activity, whereas methyl substitutions reduced it. Notably, substituting hydroxyl (2v) (**26**) at the four position of benzene ring B significantly boosted inhibitory activity. Esterification or amidation of carboxyl groups, or their direct removal, led to decreased activity, underscoring the importance of carboxyl groups. Compound 2v (**26**) (EC_50_ of 0.33 ± 0.10 μM, SI of 49) exhibited the strongest inhibitory activity among the designed compounds, indicating that carboxyl groups were important for increased activity and that carboxyl derivatives were more effective than esterified counterparts. This reduced activity due to carboxylate esterification was also evident in the study of HCoV-OC-43 virus inhibition (Compound 1b (**28**)). Modifying the carbon–carbon double bond between the benzene rings slightly improved inhibitory activity, and substituting benzene B with an ethyl group (2t (**35**) (EC_50_ of 1.02 ± 0.46 µM, SI of 71)) slightly increased activity, suggesting that benzene B was not essential for anti-HCV activity. Additionally, compound 15a (**27**) (EC_50_ of 1.42 ± 0.31 µM, SI of 113, ALogP of 3.4) showed inhibitory activity comparable to CSA though with lower hydrophobicity [117].

### 3.4. Studies on the SARs of Anti-Inflammatory Activity

Derivatives 5c (**18**), 5e (**33**), and 5h (**34**) (20 µM with inhibition rates of 60.59 ± 13.47%, 49.79 ± 4.92%, and 47.21 ± 10.74%, respectively) showed stronger inhibition of NO release compared to CSA, indicating that compounds with halogen substituents on phenyl ring B had enhanced anti-inflammatory effects. Cajanuslactone (**10**) markedly decreased the production of LPS-induced proinflammatory mediators both in vitro and in vivo. Its stronger inhibitory effect on TNF-α and IL-1β could be attributed to having a single free phenol hydroxyl group and a methoxy group at position 5 or 7, which likely enhances protection against degradation during pharmacodynamic processes. Therefore, cajanuslactone holds the potential for development as an effective drug for treating and preventing inflammation or related diseases [141].

## 4. Total Synthesis of CSA

**Scheme 2:**
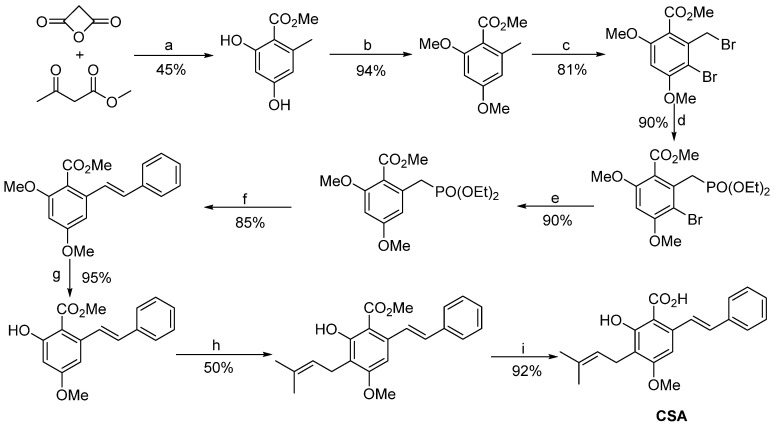
Reagents and conditions: (a) NaH, THF; (b) MeI, K_2_CO_3_, acetone, 60 °C; (c) NBS, BPO, CCl_4_, reflux; (d) P(OEt)_3_, 150 °C; (e) Pd(OAc)_2_, PPh_3_, K_2_CO_3_, *n*-BuOH, 100 °C; (f) NaH, PhCHO, THF; (g) BCl_3_, −78 °C; (h) NaH, toluene, prenyl bromide, 40 °C; (i) KOH, EtOH, H_2_O, reflux.

Since the extraction ratio is low, many research groups conducted the total synthesis of CSA. Here, we also reviewed the synthetic routes and compared each other. In 2011, Xing Yue Ji et al. successfully obtained CSA via nine steps with an overall yield of 10.3% (Scheme 2) [29]. The key step in this route is Horner–Wadsworth–Emmons (HWE) reaction to obtain E-type olefin. The highest temperature is 150 °C and phosphine reagent which is unfriendly to the environment is used. Therefore, this strategy is unfeasible for large-scale CSA production.

**Scheme 3:**
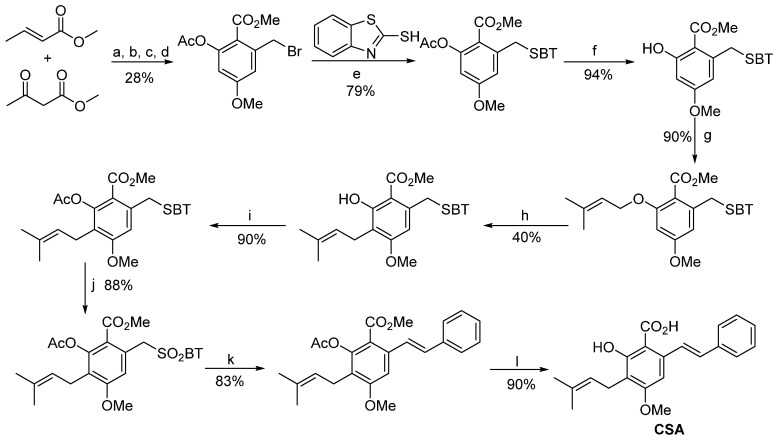
Reagents and conditions: (a) MeONa, MeOH, reflux; (b) I_2_, MeOH, reflux; (c) AcO, TEA, DMAP, DCM, r.t.; (d) NBS, BPO, CCl_4_, reflux; (e) TEA, DCM, r.t.; (f) K_2_CO_3_, MeOH, r.t.; (g) prenyl bromide, NaH, DMF, 0 °C to r.t.; (h) Montmorillonite K10, DCM, r.t.; (i) TEA, DCM, r.t.; (j) Na_2_WO_4_·2H_2_O, H_2_O_2_, MeOH; (k) NaH, ArCHO, DMF; (l) NaOH, THF/H_2_O.

In 2014, Indrapal S. Aidhen et al. reported a common building block for the syntheses of CSA (Scheme 3) [154]. In this method, the key intermediate bromine was obtained in four steps with a low yield of 28%. Two steps of acetyl protection and deprotection are required. CSA was synthesized in twelve steps with a low yield of 4.4%, which is also unfeasible for large-scale CSA production.

**Scheme 4:**
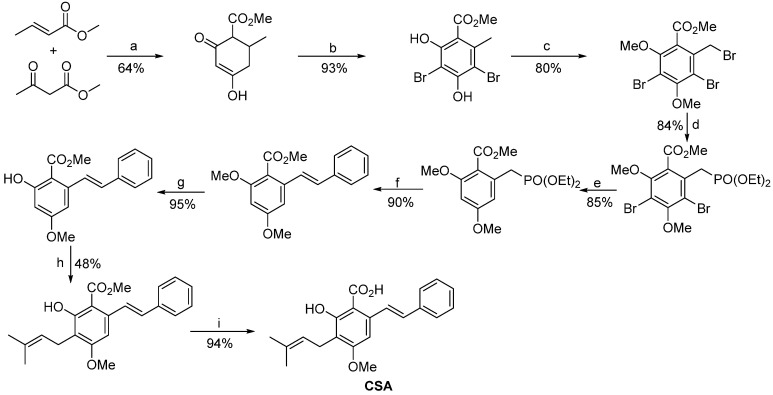
Reagents and conditions: (a) MeONa, MeOH, reflux; (b) Br_2_, AcOH, r.t.; (c) MeI, K_2_CO_3_, acetone, reflux; NBS, BPO, CCl_4_, reflux; (d) P(OEt)_3_, 150 °C; (e) Pd(OAc)_2_, PPh_3_, K_2_CO_3_, *n*-BuOH, 100 °C; (f) NaH, PhCHO, THF; (g) BCl_3_, −78 °C; (h) NaH, toluene, prenyl bromide, 40 °C; (i) KOH, EtOH, H_2_O, reflux.

In 2015, Zhi-Zhong Geng et al. reported the design, synthesis, and biological evaluation of CSA derivatives (Scheme 4) [131]. They synthesized CSA in nine steps with a yield of 13.1%, which is higher than that reported by Xing Yue Ji et al. They also used the HWE reaction to build trans-stilbene. The isopentenylation of carbon is the key reaction in this scheme. Bromine is used in the oxidative aromatization step, followed by dibromination with palladium catalyst. This is uneconomical and the methyl iodide used in the protection process is toxic.

**Scheme 5:**
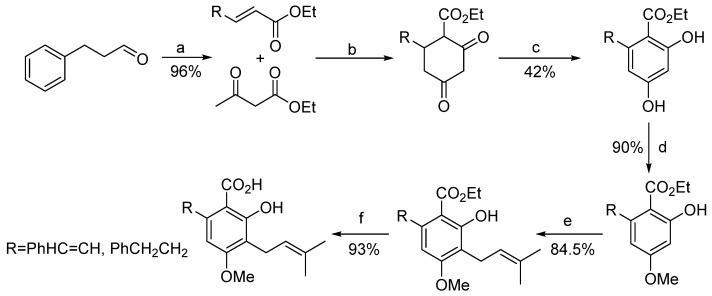
Reagents and conditions: (a) triethyl phosphonoacetate, K_2_CO_3_, PEG-400 (cat.), DCM, r.t.; (b) base, THF; (c) Br_2_, MeOH, reflux; (d) dimethyl sulfate, K_2_CO_3_, PEG-400 (cat.), acetone, 50 °C; (e) NaH, toluene, prenyl bromide, 40 °C; (f) NaOH, THF/H_2_O.

All the above-mentioned strategies are common in the construction of trans-stilbenes by alkenization reaction. In addition, we can also synthesize CSA by the aromatic ring construction strategy. For example, Yao Yao Song et al. reported the total synthesis of amorfrutin A and CSA in five steps with an overall yield of 27.2% (Scheme 5) [155]. Acrylates and acetoacetate compounds were used as starting materials. Michael addition and aldol condensation reactions were employed. Though the yield is higher than the above-mentioned three methods, the use of highly toxic mercury acetate and dimethyl sulfate promotes safety concerns.

**Scheme 6:**
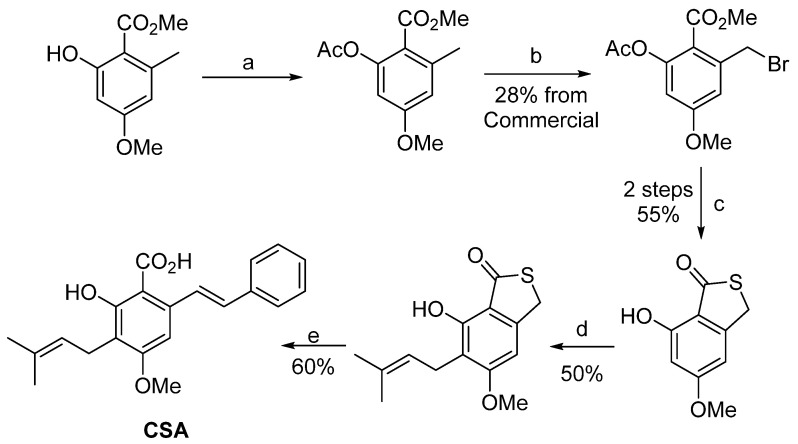
Reagents and conditions: (a) Ac_2_O, TEA, DMAP, DCM; (b) NBS, BPO, CCl_4_, reflux; (c) S=C(NH_2_)_2_, Me_2_CO, reflux; NaHCO_3_, H_2_O; (d) 2-methylbut-3-en-2-ol, amberlyst-15, DCE, reflux; (e) benzylaldehyde, LTB, −60 °C to r.t.

All the methods mentioned above are uneconomic or considered unsafe. Prithiba Mitra et al. reported a method in which the initial material was protected by acetylation and then synthesized CSA by the benzyl bromide reaction (Scheme 6) [156]. The route is short and safety because strong base for ester hydrolysis is unneeded. But the starting material is not commercially available.

**Scheme 7:**
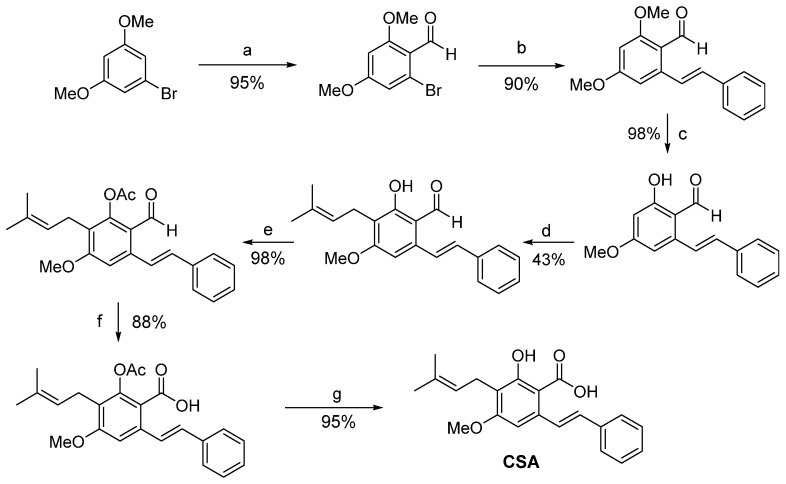
Reagents and conditions: (a) NaH, *n*-BuLi, THF, 0 °C to r.t.; (b) DIPA, *n*-BuLi, TMSCl, THF, −78 °C to r.t.; (c) TiCl_4_, DCM, −78 °C to r.t.; (d) MeONa, MeOH; (e) ArCHO, TiCl_4_, Zn, THF, reflux; (f) KOH, EtOH/H_2_O; (g) reflux. K_2_CO_3_, MeOH, r.t.

In 2018, Xing Jun Xu et al. reported the synthesis of CSA via seven steps with a yield of 29.5%, as indicated in Scheme 7 [122]. In this strategy, non-toxic agents are used and the yield is higher than all the mentioned methods.

**Scheme 8:**
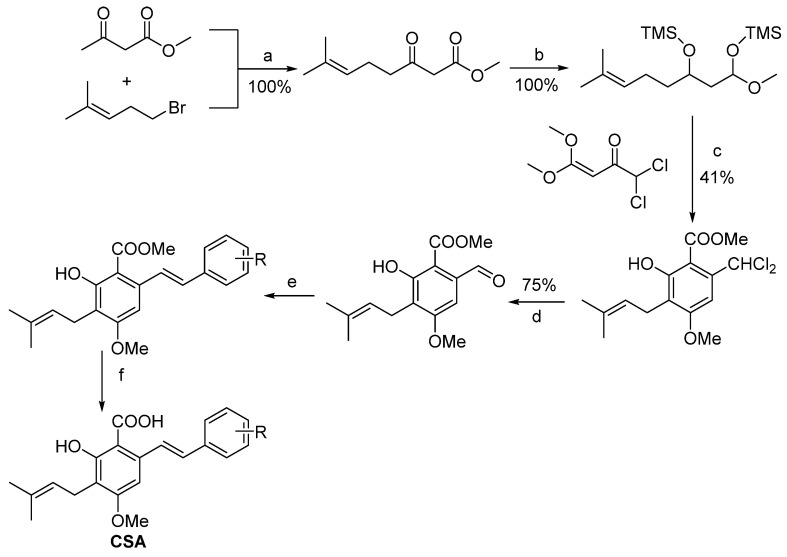
Reagents and conditions: (a) POCl_3_, DMF, 100 °C, 4 h; (b) styrene, Pd(OAc)_2_, DMA, K_3_PO_4_ 120 °C, 4 h; (c) BCl_3_ 0 °C, 2 h; (d) 3,3-Dimethylallyl bromide, NaH, toluene, 65 °C, 4 h; (e) Ac_2_O, TEA, DMAP, DCM, 0 °C to r.t. 1 h; (f) NaClO_2_, NaH_2_PO_4_, 2-methyl-2-butene, t-BuOH/H_2_O/THF.

In 2019, Qi Chen et al. reported a six steps route to synthesize CSA with an overall yield of 20% starting from commercial materials, where the key steps include TiCl_4_-mediated [3 + 3] cyclization and McMurry coupling (Scheme 8) [157]. Though the yield is lower than that reported by Xing Jun Xu et al.’s report [122], the route is shorter.

**Scheme 9:**
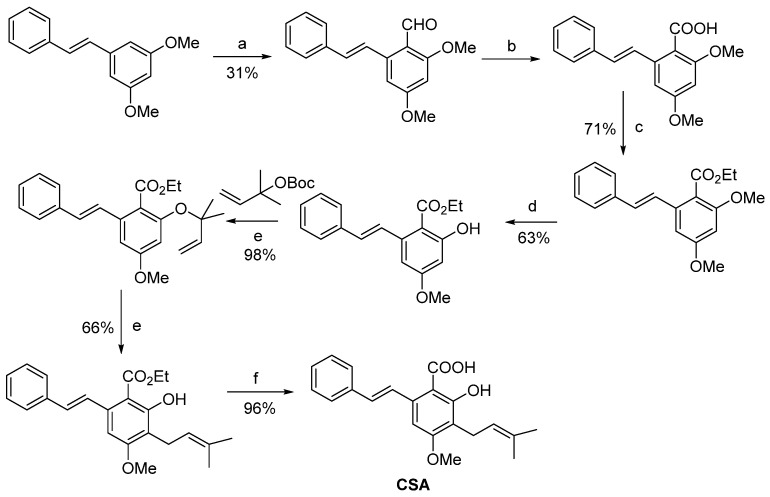
Reagents and conditions: (a) POCl_3_, DMF, −4 °C to r.t.; (b)NaClO_2_, NaH_2_PO_4_, 2-methyl-2-butene THF/H_2_O (10:1), r.t.; (c) EtI, K_2_CO_3_, MeCN, 50 °C; (d) BBr_3_, DCM, −78 °C; (e) Pd(PPh_3_)_4_, 4 Å MS, THF, −20 °C; (f) toluene, reflux; (e) KOH, EtOH/H_2_O (1:1), 80 °C.

In 2022, Tadafumi Fujita and co-workers reported an original synthetic route to CSA with a yield of 8.6% (Scheme 9) [33] which is low than all the previous reported methods. Therefore, this method is unfeasible for large amount of CSA production. In a word, eight total synthesis methods are reported by different research groups. The methods reported by prof. Chen group are more safety with high yields [122,157].

## 5. Conclusions and Discussion

In summary, CSA and its derivatives display diverse biological activities, including antibacterial, anti-inflammatory, antitumor, and so on. They may have utility in various diseases such as osteoporosis, tumors, inflammatory injuries, Alzheimer’s disease, depression, multidrug-resistant bacteria, hyperlipidemia, and ischemic stroke. But there are no studies of other diseases (such as arrhythmia, hypnosis, hypertension, and so on) related to these compounds. The structures and anti-pathogenic microorganism relationships of these compounds are also widely studied by various research groups. As a whole, the isoprene group, adjacent to the hydroxyl group, free carboxyl group, and styrene group on the benzene ring of CSA is essential for the antibacterial activity of CSA [131]. The electron-withdrawing substituent groups (such as trifluoromethyl, fluorine, and chlorine) on the B ring and geranyl in the A ring of CSA can elevate the anti-microbial activity, while the electron-donating groups are unfavored. If the B ring is replaced by a substituted p-phenylphenyl group, the anti-bacteria activity is elevated. The isopentene group on the A ring and the B ring are not necessary for the anti-HCV activity. The structures and anticancer relationships are seldom revealed. The bioactivities are largely mediated through the regulation of key cell signaling pathways, enzymes, and factors such as NF-κB, PI3K/Akt/mTOR, Nrf2/ARE, AMPK, and MAPK. These pathways serve as primary functional targets or signaling mechanisms modulated by CSA. There are eight total synthesis strategies, among which, the strategies put forward by Prof. Chen’s group are more feasible.

Currently, there is an urgent need to investigate the genes and binding receptors of CSA and to further explore its pharmacological activities, mechanisms, and therapeutic targets for specific diseases as described by Kuo Lu et al. [118]. High-throughput proteomics technology can be employed to study the intrinsic and direct targets of CSA, shedding light on its mode of action [158]. There is an urgent need to develop novel dosage forms that ensure stable drug release and clinical suitability. Alternatively, modifying CSA’s structure to overcome its drawbacks, such as poor solubility, could broaden its clinical applications and improve drug efficacy. Using nanotechnology holds promise for enhancing the delivery of bioactive compounds and pharmaceutical formulations, thereby advancing the clinical applications of CSA. Furthermore, CSA enhances drug efficacy through synergistic interactions with other medications. Exploring strategies to strengthen these adjunctive effects of CSA also represents a promising direction for future research. Last but not least, due to its limited availability and challenging extraction process from pigeon pea, obtaining a substantial quantity of CSA is constrained by both time and space [24]. Therefore, there is a pressing need to develop new methods for scaling up CSA production. This includes enhancing the extraction process from pigeon pea to improve yield [159], devising more efficient chemical synthesis methods [157], exploring biomimetic synthesis as an alternative to plant-derived CSA [149], and employing co-cultivation of pigeon pea hairy root cultures with live *Aspergillus* fungi to facilitate CSA production [160].

In conclusion, CSA and its derivatives remain promising subjects for extensive research, particularly in the realms of antibacterial and antitumor therapeutics. Further investigations into the molecular mechanisms of CSA, including structural modifications, hold the potential to enhance its clinical applicability and utility. We advocate for continued exploration and study of CSA to uncover new insights that could lead to improved treatments. With advancements in combinatorial chemistry, rational drug design, and chemical genomics, we anticipate the discovery of more CSA derivatives with robust biological activity and broad application prospects by researchers worldwide.

## Figures and Tables

**Table 1:** Molecules or signaling pathways involved in the antitumor activity of CSA.

Type	Cell Lines	In Vivo Mode	Target/Pathway	Effect	Ref.
Breast cancer	ERα-negative MDA-MB-231 (IC_50_ of 175.76 ± 19.59 μM), ERα-positive MCF-7 (IC_50_ of 61.25 ± 2.67 μM). Tamoxifen-resistant MCF-7 (MTR-3) (IC_50_ of 188.22 ± 38.58 µM)	Nude mice (dosage: 15 and 30 mg/kg; route: subcutaneous injection; cycle: 1 time/2 days, 7 times).	ERα, p53.	Inhibit growth.	[24]
	MCF-7 (8.88–14.79 μM).	None reported.	Caspase 3, Bax, p21; BRCA-1/2, BcL-2.	Induce cell cycle arrest and apoptosis.	[25]
	MCF-7 (IC_50_ of 42 to >80 µg/mL).	None reported.	None reported.	Reduce viability.	[26]
	MCF-7 (EC_50_ of 15.48 ± 6.84 μM)	None reported.	None reported.	Reduce viability.	[27]
Cervical cancer	HeLa (IC_50_ of 39 to >80 µg/mL).	None reported.	with IC_50_ values of 39~80 µg m/L, 32~80 µg m/L.	Reduce viability.	[26]
	HeLa (IC_50_ of 44.9~78.3 μM).	None reported.	None reported.	Inhibit proliferation.	[28]
Liver cancer	Bel-7402 (IC_50_ of 19.14 μM).	None reported.	None reported.	Inhibit proliferation.	[29]
Human colorectal adenocarcinoma	CaCo-2 (IC_50_ of 32~80 µg/mL).	None reported.	None reported.	Reduce viability.	[26]
	SW480 (IC_50_ of 44.9~78.3 μM).	None reported.	None reported.	Inhibit proliferation.	[28]

**Table 2:** Structures of natural derivatives of CSA.

Structures	Source	References
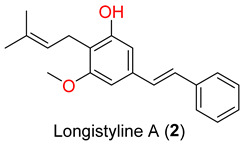	Isolated natural	[30]
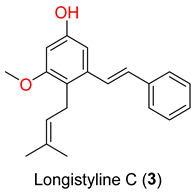	Isolated natural	[28]
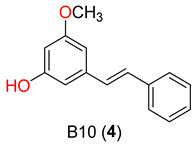	Isolated natural	[31,32]
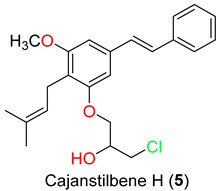	Isolated natural	[33]

**Table 3:** Structures of synthetic CSA derivatives which displayed antitumor activities.

Structures	Source	References
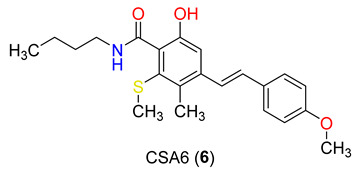	Synthetic	[27]
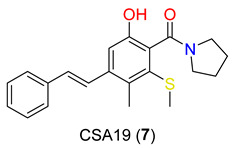	Synthetic	[27]
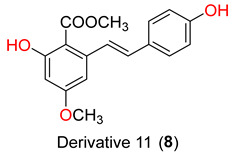	Synthetic	[49]

**Table 4:** Molecules or signaling pathways involved in the antitumor activity of CSA derivatives.

Compounds	Cell Lines	Target/Pathway	Effect	Ref.
Longistylin A (**2**) and Longistylin C (**3**)	MCF-7, COR-L23, C32, HepG2, 16HBE4o, AR42J-B13 (2.4–20.04 μM and 5.8–18.3 μM, respectively).	None reported.	Reduce viability.	[30]
Longistyline A (**2**)	Bel-7402 (IC_50_ of 24.65 μM).	None reported.	Inhibit proliferation.	[29]
Longistylin C (**3**)	MDA-MB231, HeLa, HepG2, SW480, A549, NCI-H460 and NCI-H1299 (IC_50_ of 14.4–29.6 μM).	None reported.	Inhibit proliferation.	[28]
B10 (**4**)	Raji cells (12, 18 and 24 µM, IC_50_ of 18 µM).	P-γ-H2A.X, Bax/BcL-2, cl- caspase 3, cl-caspase 9, cl-PARP1, APAF-1, JAK2/STAT3, KRAS/HDAC1/EP300/PEBP1.	Induce cell cycle arrest and apoptosis; inhibit proliferation.	[32]
Cajanstilbene H (**5**)	NCI-H460, PC-3, MCF-7, HeLa, HCT-15, and KB-V1 (IC_50_ of 21.42–25.85 μM).	None reported.	Inhibit proliferation.	[42]
CSA 6 (**6**)	MCF-7 (IC_50_ of 2.96 μM).	None reported.	Reduce viability.	[27]
CSA 6 (**6**) and CSA 19 (**7**)	CSCs-rich MCF-7 cells (IC_50_ of 4.98 and 7.51 μM, respectively).	None reported.	Reduce viability; increased collateral sensitivity.	[55]
Derivative 11 (**8**)	HT29, MCF-7 and PA-1 (IC_50_ of 56.07, 115.85 and 26.80 μM, respectively).	ERα.	Inhibit migration and invasion.	[56]

**Table 5:** Molecules or signaling pathways targeted by the systemic and organ protection activity of CSA and its derivatives.

Type	Cell Lines/In Vivo Mode	Target/Pathway	Effect	Ref.
Neuroprotective	Mice (dosage: sECC of 200 mg/kg; route: orally; cycle: 1 time/day, 7 days).	SOD, ChAT.	Improve spatial learning ability.	[13]
	Mice (dosage: 7.5, 15 and 30 mg/kg; route: intragastric administration; cycle: 1 time/day, 20 days).	Aβ_1–42_, GluN 2B; PKA/CREB/BDNF/TrkB.	Improve cognitive function.	[64]
	PC12 cells (2–16 μM).	LDH, Ca^2+^, Caspase 3, Cl-caspase 3.	Increase cells viability.	[62]
	PC12 cells (1–8 μM).	ROS, MDA, GRP 78, CHOP/GADD 153, XBP-1, Caspase 12, Caspase 9; SOD, CAT.	Increase cells viability.	[63]
	Mice (dosage: 7.5, 15 and 30 mg/kg; route: intragastric administration; cycle: 1 time/day, end of experiment).	NE, DA, GABA, Glu, ACh.	Improve depression-like behavior.	[71]
	Mice (dosage: 4, 7.5, 15, 30, 60 and 120 mg/kg; route: intragastric administration; cycle: 1 time/day, 7 days.)	KP, KYNA, 5-HT, TRP, BDNF, PSD-95, Akt/mTOR.	Improve depression-like behavior.	[77]
	SH-SY5Y cells (0.1–1 μM).	ROS, MDA; SOD, AMPK/Nrf 2.	Reduce cell apoptosis and improve oxidative stress.	[81]
Cardiocerebral vascular system	Mice (dosage: sECC of 100 and 200 mg/kg; route: intragastric administration; cycle: 1 time/day, 28 days).	LDL; SOD, HMG-CoA mRNA, CYP7A1 mRNA, LDLR mRNA.	Reduce cholesterol.	[87,88]
	HepG 2 (MECC of 0.05 and 0.1 mg/mL).	PCSK 9, LDL.	Reduce cholesterol.	[18]
	VSMC (0.1–10 μM)	PKC, and Rho/Rho, Ca^2+^.	Dilate the renal arteries.	[92]
	H9c2	ROS, MDA, Ca^2+^, Bax/BcL-2; SOD, CAT, MMP, caspase-3.	Increase cells viability.	[95]
Cartilage protection	Rat (dosage: 50, 100 and 200 mg/kg; route: intragastric administration; cycle: 1 time/day, 6 days/week, for 8 consecutive weeks).	FSH, LH.	Inhibit bone loss.	[98]
	HOS TE85 (0.001, 0.01, 0.1 μg/mL).	None reported.	Inhibit bone loss.	[99]
	Rats (dosage of sECC: 0.8 and 8 g/kg; route: intragastric administration; cycle: 1 time/day, 14 days).	None reported.	Inhibit bone loss.	[100]
	BMM (2.5–10 μM).	ROS, NFATc1, C-Fos, V-ATPase-d1/2, NF-κB.	Inhibit bone loss and the activity of osteoclasts.	[102]
	hMSC (cajanstilbene H (5) 1–4 μM).	None reported.	Promote osteoblast differentiation.	[42]
Immunoregulation	NK cells (LJ101019C (9) 0.1–1 µM).	ROS, Kv1.3, AKT/mTOR.	Promote cells proliferation and improve immunity.	[107]
Liver protection	Mice (dosage: 50 and 75 mg/kg; route: orally; cycle: only 1 time)	IFN-γ, TNF, iNOS, IL-1β, p-IκBα, p-p65, p62; PGC-1α, TFAM, LC3-II, PINK1, Parkin, Sestrin 2/LKB 1/AMPK.	Reduce oxidative stress.	[112]

**Table 6:** Structures of CSA derivatives which displayed anti-microbial activities.

Structures	Source	References
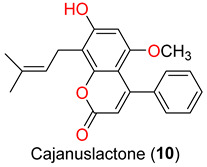	Isolated natural	[115]
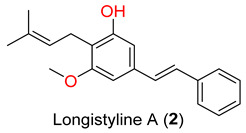	Isolated natural	[116]
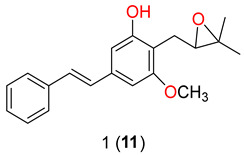	Isolated natural	[117]
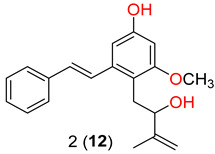	Isolated natural	[117]
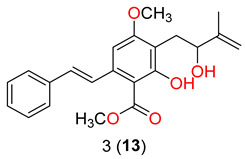	Isolated natural	[117]
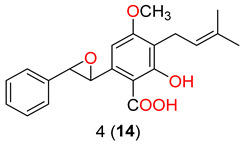	Isolated natural	[117]
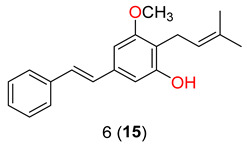	Isolated natural	[117]
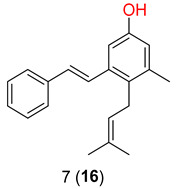	Isolated natural	[117]
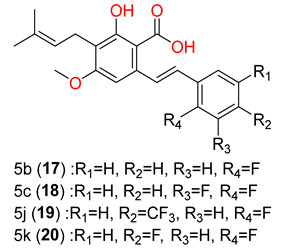	Synthetic	[118]
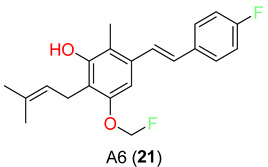	Synthetic	[119]
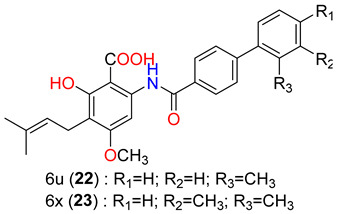	Synthetic	[120]
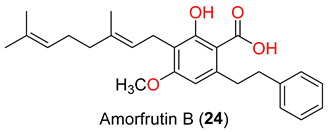	Synthetic	[121]
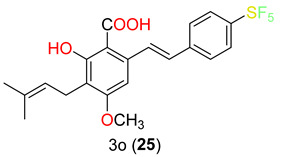	Synthetic	[122]
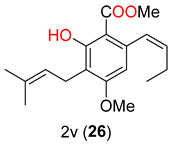	Synthetic	[15]
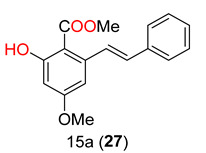	Synthetic	[15]
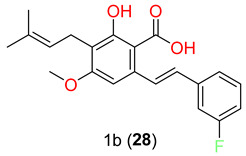	Synthetic	[123]

## Data Availability

No new data were created or analyzed in this study.
